# mGluR5/ERK signaling regulated the phosphorylation and function of glycine receptor α1^ins^ subunit in spinal dorsal horn of mice

**DOI:** 10.1371/journal.pbio.3000371

**Published:** 2019-08-21

**Authors:** Zi-Yang Zhang, Hu-Hu Bai, Zhen Guo, Hu-Ling Li, Yong-Tao He, Xing-Lian Duan, Zhan-Wei Suo, Xian Yang, Yong-Xing He, Xiao-Dong Hu

**Affiliations:** 1 Department of Molecular Pharmacology, School of Pharmacy, Lanzhou University, Lanzhou, Gansu, PR China; 2 School of Life Sciences, Lanzhou University, Lanzhou, Gansu, PR China; University/ETH Zürich, SWITZERLAND

## Abstract

Inhibitory glycinergic transmission in adult spinal cord is primarily mediated by glycine receptors (GlyRs) containing the α1 subunit. Here, we found that α1^ins^, a longer α1 variant with 8 amino acids inserted into the intracellular large loop (IL) between transmembrane (TM)3 and TM4 domains, was expressed in the dorsal horn of the spinal cord, distributed at inhibitory synapses, and engaged in negative control over nociceptive signal transduction. Activation of metabotropic glutamate receptor 5 (mGluR5) specifically suppressed α1^ins^-mediated glycinergic transmission and evoked pain sensitization. Extracellular signal-regulated kinase (ERK) was critical for mGluR5 to inhibit α1^ins^. By binding to a D-docking site created by the 8-amino–acid insert within the TM3–TM4 loop of α1^ins^, the active ERK catalyzed α1^ins^ phosphorylation at Ser380, which favored α1^ins^ ubiquitination at Lys379 and led to α1^ins^ endocytosis. Disruption of ERK interaction with α1^ins^ blocked Ser380 phosphorylation, potentiated glycinergic synaptic currents, and alleviated inflammatory and neuropathic pain. These data thus unraveled a novel, to our knowledge, mechanism for the activity-dependent regulation of glycinergic neurotransmission.

## Abstract 2

Activity-dependent phosphorylation of the glycine receptor α1ins subunit by metabotropic glutamate receptor 5 and ERK kinase signalling causes endocytosis of α1ins and glycinergic disinhibition in the spinal cord dorsal horn, contributing to pain sensitization.

## Introduction

Glycine receptors (GlyRs) are ligand-gated chloride channels that mediate fast inhibitory synaptic transmission in the spinal cord, retina, brain stem, and other brain regions [1,2]. Glycinergic inhibition plays an important role in the modification of motor and sensory functions [1–3]. Four α subunits (α1–α4) and one β subunit have, to date, been identified to form pentameric GlyRs [4]. These subunits display spatiotemporal distribution and assign distinct biological properties to GlyRs. The protein level of the α2 subunit in the spinal cord declines rapidly after birth [2,4]. In adulthood, the vast majority of glycinergic transmission is generated by those GlyRs containing the α1 subunit [4,5]. The GlyR α3 subunit also contributes to spinal glycinergic transmission, albeit to a lesser extent than α1 [5]. Alternative splicing can generate two variants for each α subunit [2]. α1^ins^ is a longer variant of the α1 subunit with 8 amino acids inserted into the intracellular large loop (IL) between transmembrane (TM)3 and TM4 domains [6]. α1^ins^ is expressed in the adult spinal cord and brain stem and is estimated to account for more than 30% of total α1 subunit [6]. However, the functional significance of this splice variant remains to be elucidated.

Glycinergic transmission in the spinal cord dorsal horn negatively controls the excitability and responsiveness of nociceptive neurons. The reduced glycinergic inhibition is widely considered as a key contributor to central sensitization of nociceptive behaviors [7]. The activity-dependent regulation of GlyRs number on plasma membrane and postsynaptic sites represents an important way to modulate inhibitory synaptic strength and plasticity [8]. The intracellular trafficking process of GlyRs is precisely controlled by posttranslational modifications. Protein kinases have been shown to phosphorylate GlyRs [9–11], which either depresses or boosts glycinergic currents through mechanisms involving the altered receptor endocytosis, exocytosis, and lateral diffusion on plasma membrane [9–12]. In addition to phosphorylation, ubiquitination has also been implicated in the modification of endocytosis and surface expression of GlyRs [13].

Group I metabotropic glutamate receptors (mGluRs) are Gq/11-protein–coupled receptors that include two subtypes, mGluR1 and mGluR5. Activation of Group I mGluRs triggers multiple intracellular signaling cascades and causes long-lasting changes of excitatory and inhibitory synaptic transmission in several brain regions [14]. mGluR-dependent synaptic plasticity correlates with a number of neuropsychiatric disorders such as depression, anxiety, schizophrenia, and pathological pain [14]. Here, we found that brief stimulation of mGluR5 in spinal cord dorsal horn suppressed glycinergic transmission through extracellular signal-regulated kinase (ERK)-dependent phosphorylation and ubiquitination of the GlyR α1^ins^ variant. We provided evidence that the removal of α1^ins^-mediated glycinergic inhibition contributed to inflammatory pain.

## Results

### Activation of mGluR5 attenuated glycinergic transmission through ERK signaling

**Fig 1:**
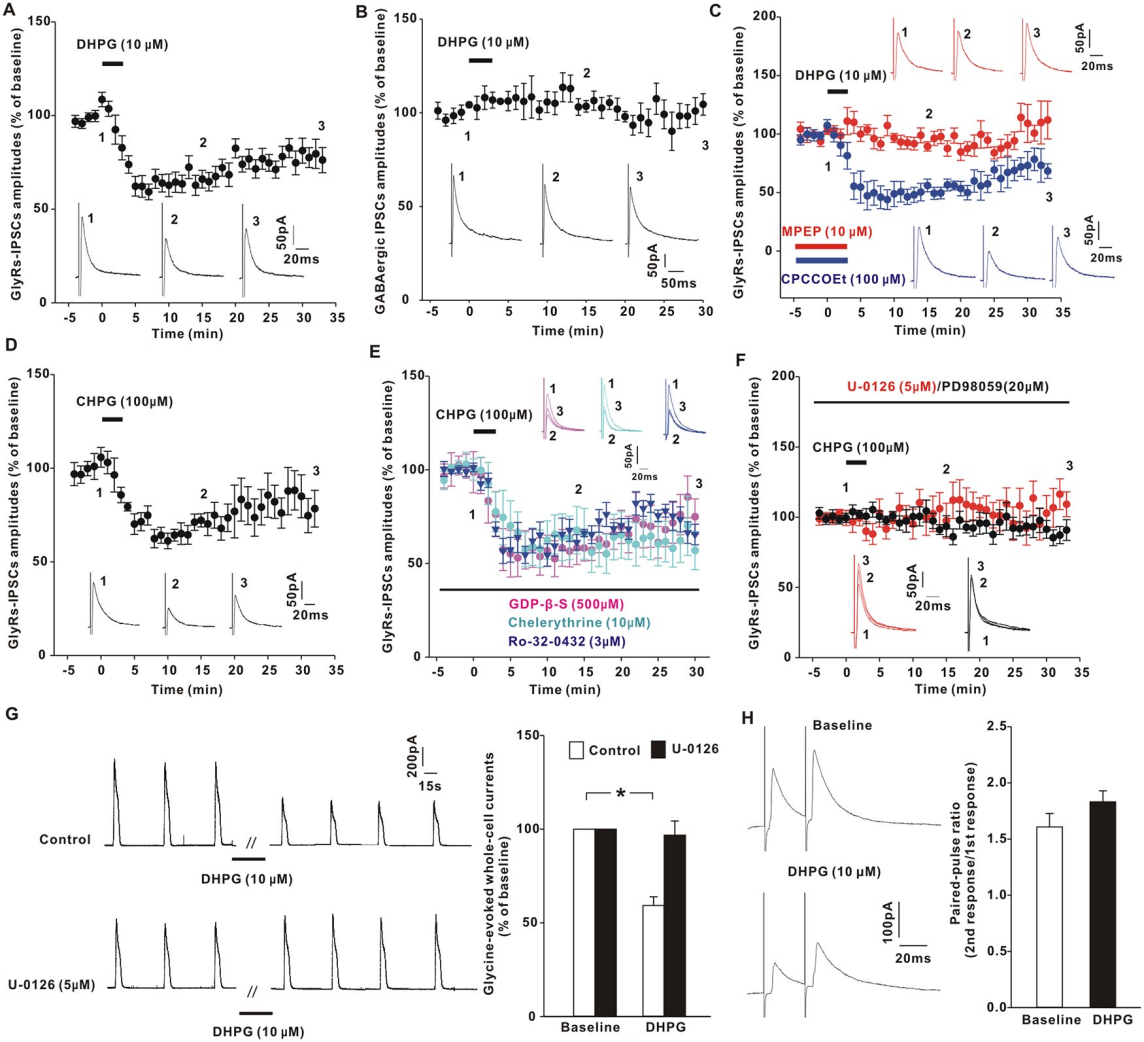
mGluR5 receptor decreased glycinergic currents. (**A–B**) Bath perfusion of DHPG for 3 min reduced GlyR-IPSCs (**A**; 70.2 ± 4.2% of baseline at 15–20 min post-DHPG, *t*[16] = 6.707, *p* < 0.001, paired Student *t* test), but not GABA_A_-receptor–mediated IPSCs (**B**; 102.9 ± 3.3% of baseline at 15–20 min post-DHPG, *t*[5] = 0.347, *p* = 0.743) in spinal slices of mice. The horizontal bar indicated the period of DHPG perfusion. The original traces were taken at the time points indicated by the numbers 1–3. (**C**) The inhibitory effect of DHPG on GlyR-IPSCs was blocked by MPEP (92.4 ± 4.4% of baseline at 15–20 min post-DHPG, *t*[5] = 1.573, *p* = 0.177) but not by CPCCOEt (53.5 ± 3.7% of baseline at 15–20 min post-DHPG, *t*[5] = 14.343, *p* < 0.001). (**D**) Bath perfusion of CHPG suppressed GlyR-IPSCs (72.6 ± 5.3% of baseline at 15–20 min post-CHPG, *t*[8] = 6.51, *p* < 0.001). (**E**) The inhibitory effect of CHPG on GlyR-IPSCs was not blocked by intracellularly loaded GDP-β-S (62.3 ± 6.4% of baseline at 15–20 min post-CHPG, *t*[6] = 2.782, *p* = 0.032), chelerythrine (60.9 ± 11.3% of baseline at 15–20 min post-CHPG, *t*[6] = 2.705, *p* = 0.035), or Ro-32-0432 (69.9 ± 3.2% of baseline at 15–20 min post-CHPG, *t*[5] = 8.495, *p* < 0.001). (**F**) Postsynaptic loading of U-0126 or PD98059 prevented CHPG from decreasing glycinergic responses (U-0126, 107.6 ± 10.4% of baseline at 15–20 min post-CHPG, *t*[8] = 0.997, *p* = 0.348; PD98059, 93.1 ± 5.0% of baseline at 15–20 min post-CHPG, *t*[5] = 0.883, *p* = 0.418). (**G**) Effects of DHPG (10 μM, 3 min) on the whole-cell currents evoked by exogenously applied glycine (1 mM; 5 s) in the absence (control) or presence of intracellular U-0126 loading. *p < 0.001 versus baseline (*t*[10] = 5.235, paired *t* test). *n* = 11 (control) and 8 neurons (U-0126). (**H**) DHPG did not affect the paired-pulse ratios of GlyR-IPSCs (*n* = 10). The underlying data for this figure can be found in S1 Data. Error bars indicated SEM. CHPG, α-amino-2-chloro-5-hydroxybenzeneacetic acid; DHPG, (S)-3,5-Dihydroxyphenylglycine; GABA_A_ receptor, γ-Aminobutyric acid type A receptor; GDP-β-S, Guanosine 5ʹ-O-(2-Thiodiphosphate); GlyR, glycine receptor; IPSC, inhibitory postsynaptic current; mGluR5, metabotropic glutamate receptor 5; MPEP, 6-Methyl-2-(phenylethynyl) pyridine; PD98059, 2ʹ-Amino-3ʹ-methoxyflavone; Ro-32-0432, 2-{8-[(Dimethylamino)methyl]-6,7,8,9-tetrahydropyrido[1,2-a]indol-3-yl}-3-(1-methyl-1H-indol-3-yl)maleimide.

The inhibitory postsynaptic currents (IPSCs) mediated by GlyRs were recorded in lamina II neurons within the spinal cord slices of mice [15]. Extracellular application of a selective Group I mGluR agonist (S)-3,5-Dihydroxyphenylglycine (DHPG) (10 μM) for 3 min induced a rapid reduction of GlyR-IPSCs (Fig 1A). This synaptic depression was specific to glycinergic responses because the same DHPG treatment generated no changes in the amplitudes of γ-Aminobutyric acid type A (GABA_A_)-receptor–mediated IPSCs (Fig 1B). Group I mGluRs consist of two subtypes: mGluR1 and mGluR5. To determine which subtype was responsible for DHPG action, we pretreated the slices with selective mGluR1 antagonist CPCCOEt or mGluR5 antagonist 6-Methyl-2-(phenylethynyl) pyridine (MPEP) before DHPG application. CPCCOEt (100 μM) did not block the inhibitory effect of DHPG on glycinergic transmission (Fig 1C). In the presence of mGluR5 antagonist MPEP (10 μM), however, DHPG lost the ability to suppress the synaptic currents (Fig 1C). To confirm the role of mGluR5, we extracellularly applied a selective mGluR5 agonist α-amino-2-chloro-5-hydroxybenzeneacetic acid (CHPG) (100 μM), finding that CHPG perfusion for 3 min mimicked the action of DHPG by causing a significant reduction of GlyR-IPSC amplitudes (Fig 1D).

mGluR5 typically relays the signaling from Gq protein to Protein Kinase C (PKC). When G protein was inhibited by intracellular loading of nonhydrolyzable GDP analog Guanosine 5′-O-(2-Thiodiphosphate) (GDP-β-S) (500 μM) through recording pipettes, CHPG still depressed glycinergic transmission (Fig 1E). PKC inhibitor chelerythrine (10 μM) or 2-{8-[(Dimethylamino)methyl]-6,7,8,9-tetrahydropyrido[1,2-a]indol-3-yl}-3-(1-methyl-1H-indol-3-yl)maleimide (Ro-32-0432) (3 μM) also failed to block the inhibitory effect of CHPG (Fig 1E). Since mGluR5 can stimulate ERK signaling in a manner independent of Gq protein [16,17], we tested the potential role of ERK by intracellular introduction of mitogen-activated protein kinase kinase (MEK) inhibitor U-0126 (5 μM) or 2ʹ-Amino-3ʹ-methoxyflavone (PD98059) (20 μM). Our data showed that both U-0126 and PD98059 blocked CHPG from inhibiting GlyR-IPSCs (Fig 1F), suggesting that the reduction of glycinergic currents by mGluR5 was attributed to ERK activation.

The regulated synaptic transmission might result from the decrease of presynaptic glycine release or hypofunction of postsynaptic receptors. Previous studies have implicated that prolonged activation (>10 min) of mGluR1/5 by DHPG can stimulate spinal excitatory interneurons to produce endocannabinoids, which act as retrograde messengers to stimulate Type-1 cannabinoid (CB1) receptors expressed at inhibitory nerve terminals [18]. CB1 receptor activation reduces presynaptic glycine release and inhibits GlyR-IPSCs [18]. Here, we found that pretreatment with CB1 receptor antagonist AM251 (5 μM) for 30 min did not block the reduction of GlyR-IPSCs caused by brief DHPG application (S1 Fig), suggesting that short DHPG treatment was not sufficient to produce endocannabinoids. Rather, our data showed that postsynaptic loading of U-0126 or PD98059 eliminated the inhibitory effect of mGluR5 (Fig 1F), implicating a postsynaptic origin. To verify this result, we elicited the whole-cell glycinergic currents by a brief puff (5 s) of exogenous glycine (1 mM) onto the recorded neurons (Fig 1G). Extracellular application of DHPG for 3 min noticeably decreased the amplitudes of GlyR currents (Fig 1G), which were blocked by intracellularly loaded U-0126 (Fig 1G). Comparison of paired-pulse ratios of GlyR-IPSCs revealed no significant changes before and after DHPG exposure (Fig 1H), suggesting that mGluR5/ERK signaling suppressed the function or number of GlyRs on postsynaptic membrane.

### GlyR α1^ins^ subunit was the specific target for mGluR5 regulation

**Fig 2:**
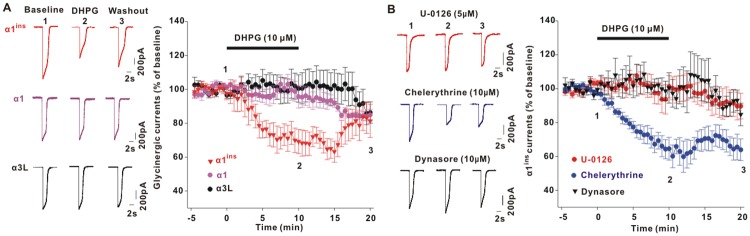
mGluR5 specifically decreased α1^ins^ currents in HEK293T cells through ERK signaling. (**A**) Effects of DHPG on glycine (1 mM, 10 ms)-induced whole-cell currents in HEK293T cells co-transfected with mGluR5a and α1^ins^ (68.3 ± 5.9% of baseline at 10–15 min post-DHPG, *t*[7] = 2.944, *p* = 0.022, paired Student *t* test), α1 (94.3 ± 4.8% of baseline at 10–15 min post-DHPG, *t*[7] = 0.138, *p* = 0.894), or α3L (101.8 ± 9.2% of baseline at 10–15 min post-DHPG, *t*[7] = 0.49, *p* = 0.639). (**B**) The inhibitory effect of DHPG on α1^ins^ currents was blocked by U-0126 (99.5 ± 7.0% of baseline at 10–15 min post-DHPG, *t*[7] = 0.032, *p* = 0.975) and dynasore (102.3 ± 10.4% of baseline at 10–15 min post-DHPG, *t*[6] = 0.714, *p* = 0.502) but not by chelerythrine (64.7 ± 5.1% of baseline at 10–15 min post-DHPG, *t*[5] = 4.487, *p* = 0.006). The underlying data for this figure can be found in S1 Data. Error bars indicated SEM. DHPG, (S)-3,5-Dihydroxyphenylglycine; ERK, extracellular signal-regulated kinase; HEK, human embryonic kidney; mGluR5, metabotropic glutamate receptor 5.

Glycinergic inhibition in the adult spinal cord is predominantly produced by GlyRs with α1 subunit [2,5]. Alternative splicing of *GLRA1* transcript can generate a longer variant, α1^ins^ [6]. To test which of the functional GlyRs subunits was regulated by DHPG, we recorded glycine-activated whole-cell currents in human embryonic kidney (HEK)293T cells coexpressing individual GlyR subunit and mGluR5a. Puffer application of glycine (1 mM, 10 ms) evoked robust membrane currents in cells expressing either α1 or α1^ins^ (Fig 2A). Activation of mGluR5a by DHPG did not affect the peak currents mediated by α1 (Fig 2A). However, α1^ins^ currents were potently inhibited by DHPG (Fig 2A). As was seen in spinal slices, intracellular loading of MEK inhibitor U-0126 (5 μM; Fig 2B) or PD98059 (20 μM; S2 Fig) blocked DHPG from depressing α1^ins^ currents. In the presence of PKC inhibitor chelerythrine (10 μM; Fig 2B) or Ro-32-0432 (3 μM; S2 Fig), however, DHPG still caused α1^ins^ inhibition. The mGluR5/ERK signaling might take effect by inducing α1^ins^ endocytosis. When the cells were pretreated with dynamin inhibitor dynasore (10 μM), no reduction of α1^ins^ currents was observed in response to DHPG challenge (Fig 2B). We also investigated the effect of DHPG on GlyR α3L subunit, a longer α3 variant involved in pain modification [19], and found no reduction of α3L currents after DHPG exposure (Fig 2A). These data suggested that mGluR5 specifically inhibited α1^ins^ function.

### Role of α1^ins^ in spinal nociceptive processing

**Fig 3:**
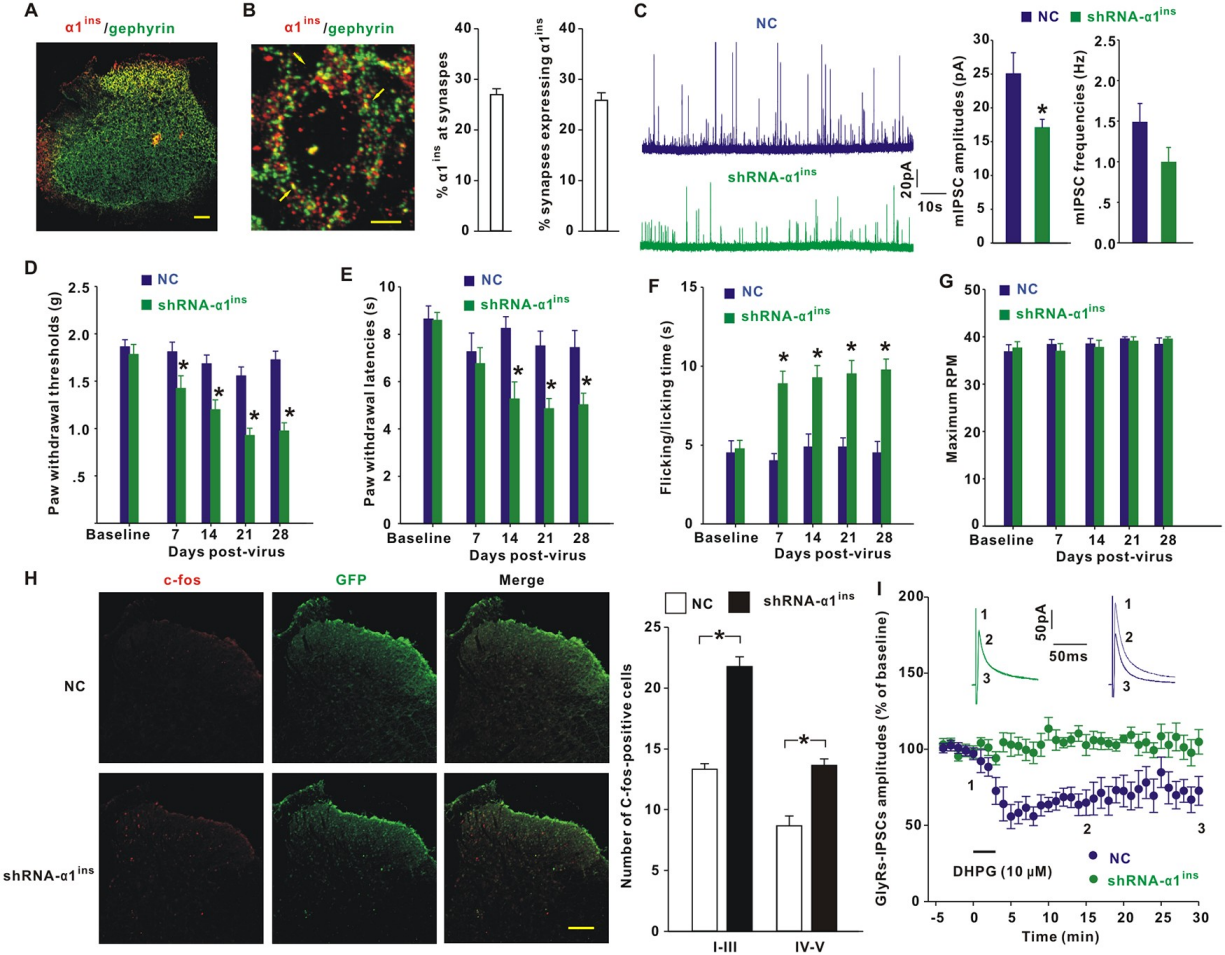
Role of α1^ins^ in spinal nociceptive modification. (**A**) Immunohistochemical analysis of α1^ins^ (red) and gephyrin (green) expression in the spinal cord of mice. Scale bar: 100 μm. (**B**) α1^ins^ and gephyrin puncta in the superficial dorsal horn at high magnification. The arrows indicated α1^ins^ colocalization with gephyrin. The graphs showed the percent of α1^ins^ puncta at synapses (synaptic α1^ins^/total α1^ins^ puncta) and percent of synapses expressing α1^ins^ (synaptic α1^ins^/total gephyrin puncta). *n* = 21 slices from 5 mice. Scale bar: 5 μm. (**C**) GlyR-mIPSC amplitudes were decreased at day 28 after intraspinal injection of AAV encoding shRNA-α1^ins^. *p = 0.038 versus NC (Mann–Whitney U test). *n* = 8 neurons/group. (**D–F**) shRNA-α1^ins^ elicited mechanical allodynia (**D**, F[4, 72] = 3.268, *p* = 0.016, repeated measures ANOVA, *n* = 10 mice/group), heat hyperalgesia (**E**, F[4, 56] = 3.405, *p* = 0.015, *n* = 8 mice/group), and cold hyperalgesia (**F**, F[4, 56] = 3.733, *p* = 0.009, *n* = 8 mice/group). *p < 0.05 versus NC (post hoc Bonferroni test). (**G**) shRNA-α1^ins^ did not change the maximum tolerated RPM in accelerating rotarod test (F[4, 56] = 0.536, *p* = 0.71, *n* = 8 mice/group). (**H**) c-fos expression at day 28 after NC or shRNA-α1^ins^ injection. *p < 0.001 versus NC (Mann–Whitney U test). *n* = 12 sections/group. Scale bar: 100 μm. (**I**) shRNA-α1^ins^ blocked DHPG from reducing GlyR-IPSCs (104.5 ± 3.7% of baseline at 15–20 min post-DHPG, *t*[12] = 1.8, *p* = 0.097, paired Student *t* test), while NC had no effect (69.3 ± 9.2% of baseline at 15–20 min post-DHPG, *t*[8] = 2.774, *p* = 0.024). The underlying data for this figure can be found in S1 Data. Error bars indicated SEM. AAV, adeno-associated virus; ANOVA, Analysis of Variance; DHPG, (S)-3,5-Dihydroxyphenylglycine; GFP, green fluorescent protein; GlyR, glycine receptor; IPSC, inhibitory postsynaptic current; mIPSC, miniature IPSC; NC, negative control shRNA; RPM, rounds per minute; shRNA, short hairpin RNA.

To date, little is known about the biological properties of α1^ins^. To address this issue, we developed a rabbit antibody against α1^ins^ (anti-α1^ins^). In transfected HEK293T cells, anti-α1^ins^ antibody recognized α1^ins^ but not α1 or α3L (S3A Fig). A western blot also illustrated a specific reaction of the antibody with α1^ins^ (S3B Fig). By using this custom-made antibody, our data revealed abundant expression of α1^ins^ in the superficial dorsal horn of the spinal cord (Fig 3A). A fraction of α1^ins^ puncta (27.0 ± 1.2%, *n* = 21 slices) were colocalized with inhibitory synaptic marker gephyrin (Fig 3B), and 25.8 ± 1.5% of inhibitory synapses expressed α1^ins^ (Fig 3B). Preincubation with excess antigen abolished anti-α1^ins^ immunosignals (S3B and S3C Fig), confirming the specificity of the antibody.

Next, we designed a short hairpin RNA (shRNA) against mouse α1^ins^ (shRNA-α1^ins^) to reveal α1^ins^ function. In transfected HEK293T cells, shRNA-α1^ins^ specifically reduced the protein level of α1^ins^ without any influence on that of α1 (S4A Fig). We injected the adeno-associated virus (AAV) encoding green fluorescent protein (GFP) and shRNA-α1^ins^ in the dorsal horn of mice and recorded GlyR-mediated miniature IPSCs (mIPSCs) in acute slices after 28 days. GFP fluorescence was restricted to the injected side and spread rostrocaudally for about 0.5 mm (S4B Fig). Compared to negative control shRNA (NC), selective knockdown of α1^ins^ in vivo (S4C Fig) decreased the amplitudes of GlyR-mIPSCs (Fig 3C). The frequencies of GlyR-mIPSCs displayed no significant difference between shRNA-α1^ins^– and NC-injected mice (Fig 3C), suggesting a functional involvement of postsynaptic α1^ins^ in basal glycinergic transmission. Behavioral tests demonstrated that the mice developed mechanical pain hypersensitivity at days 7–28 after shRNA-α1^ins^ expression (Fig 3D). A similar sensitization was observed for heat (Fig 3E) and cold stimuli (Fig 3F) after α1^ins^ knockdown. shRNA-α1^ins^ did not cause motor impairment, as evidenced by the similar performance of NC- and shRNA-α1^ins^–injected mice on an accelerating rotarod (Fig 3G) [3]. We then examined the expression of c-fos, a marker of neuronal activation. Compared to NC, shRNA-α1^ins^ significantly increased the number of c-fos–positive soma at day 28 post-viral injection (Fig 3H), suggesting that the synaptic inhibition generated by α1^ins^ was constitutively active in the negative control over neuronal excitability.

To examine whether mGluR5 inhibited glycinergic transmission by specific down-regulation of α1^ins^ function ex vivo, we recorded GlyRs-IPSCs in neurons expressing shRNA-α1^ins^. Knockdown of α1^ins^ blocked DHPG from reducing the amplitudes of GlyRs-IPSCs (Fig 3I), while NC had no effect on the synaptic depression (Fig 3I). To confirm the specificity of DHPG in the inhibition of α1^ins^, we designed shRNA to knock down α3 subunit (shRNA-α3). Viral expression of shRNA-α3 reduced the protein level of α3 (S5A Fig), with that of α1 or α1^ins^ unaltered (S5A Fig). Similar to shRNA-α1^ins^, shRNA-α3 elicited pain sensitization to mechanical (S5B Fig), heat (S5C Fig), and cold stimuli (S5D Fig). However, in shRNA-α3–expressing neurons, DHPG still caused a significant inhibition of GlyR-IPSCs (S5E Fig).

### ERK interacted with α1^ins^

**Fig 4:**
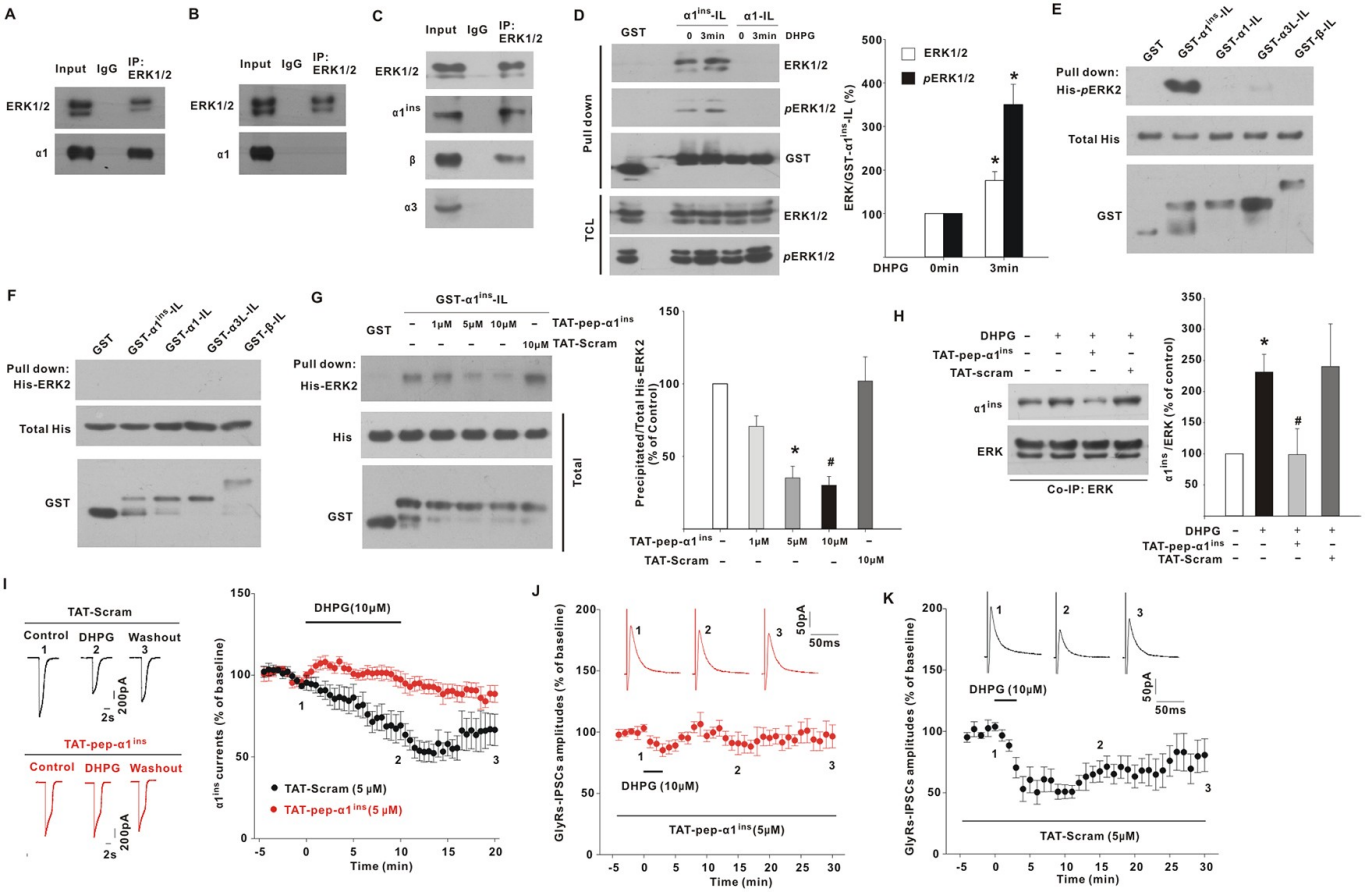
α1^ins^ interaction with ERK. (**A–B**) Co-IP with ERK1/2 antibody from lysates of HEK293T cells transfected with GlyR-α1^ins^ (**A**) or GlyR-α1 (**B**). The immunoprecipitates were probed with anti-α1 antibody. *n* = 3 experiments/group. (**C**) Co-IP with ERK1/2 antibody from spinal dorsal horn of mice. *n* = 3 experiments. (**D**) GST-α1^ins^-IL or GST-α1-IL was used to pull down ERK1/2 and *p*ERK1/2 from lysates of DHPG (10 μM; 0–3 min)-treated spinal slices. The TCLs were also immunoblotted. The graph showed the percentage changes of ERK1/2 and *p*ERK1/2 contents precipitated by GST-α1^ins^-IL. *p = 0.029 versus DHPG-untreated control (Mann–Whitney U test), *n* = 4 experiments. (**E**) The purified His-*p*ERK2 was precipitated by GST-α1^ins^-IL but not by GST-α1-IL, GST-α3L-IL, or GST-β-IL. The total His (middle) and GST proteins (bottom) were also shown. *n* = 4 experiments. (**F**) GST-α1^ins^-IL, GST-α1-IL, GST-α3L-IL, and GST-β-IL did not pull down the purified nonphosphorylated His-ERK2. *n* = 4 experiments. (**G**) TAT-pep-α1^ins^ reduced His-ERK2 contents precipitated by GST-α1^ins^-IL from lysates of HEK293T cells coexpressing MEK1(S218D/S222D). TAT-Scram was used as control. *p = 0.027, #*p* = 0.013 versus peptide-untreated control (one-way ANOVA with post hoc Bonferroni test), *n* = 6 experiments. (**H**) Intrathecal DHPG application (10 nmol, 10 min) increased α1^ins^/ERK interaction, which was blocked by pretreatment with TAT-pep-α1^ins^ (200 pmol) for 30 min. *p = 0.023 versus control, #*p* = 0.006 versus DPHG (one-way ANOVA with post hoc Bonferroni test), *n* = 6. (**I**) Intracellular loading of TAT-pep-α1^ins^ prevented DHPG from inhibiting glycine-evoked whole-cell currents in HEK293T cells coexpressing mGluR5a and α1^ins^ (93.1 ± 4.5% of baseline at 10–15 min post-DHPG, *t*[6] = 1.331, *p* = 0.231, paired Student *t* test), whereas TAT-Scram had no effect (57.3 ± 6.1% of baseline at 10–15 min post-DHPG, *t*[5] = 3.829, *p* = 0.012). (**J–K**) Postsynaptic loading of TAT-pep-α1^ins^ prevented DHPG from suppressing GlyR-IPSCs (**J**, 92.2 ± 7.1% of baseline at 15–20 min post-DHPG, *t*[9] = 1.333, *p* = 0.215, paired Student *t* test), while TAT-Scram had no effect (**K**; 67.8 ± 8.2% of baseline at 15–20 min post-DHPG, *t*[7] = 3.761, *p* = 0.007). The underlying data for this figure can be found in S1 Data. Error bars indicated SEM. ANOVA, Analysis of Variance; ERK, extracellular signal-regulated kinase; DHPG, (S)-3,5-Dihydroxyphenylglycine; GlyR, glycine receptor; GST, Glutathione S-Transferase; HEK, human embryonic kidney; IgG, immunoglobulin G; IL, intracellular large loop; IP, immunoprecipitation; IPSC, inhibitory postsynaptic current; MEK, mitogen-activated protein kinase kinase; mGluR5, metabotropic glutamate receptor 5; *p*ERK, phosphorylated ERK; TAT, human immunodeficiency virus-type 1 TAT sequence; TAT-pep-α1^ins^, TAT-fused α1^ins^-derived peptide; TAT-Scram, TAT-fused scrambled peptide; TCL, total cell lysate.

For many kinases, the full access to substrates has been deemed a key step to achieve their specificity in biological regulation. Since mGluR5 inhibited glycinergic responses through ERK, we tested whether this kinase physically interacted with α1^ins^. From lysates of transfected HEK293T cells, anti-ERK antibody precipitated α1^ins^ (Fig 4A) rather than α1 (Fig 4B), suggesting the specificity of ERK binding to α1^ins^. Coimmunoprecipitation experiments from spinal cord dorsal horn illustrated that ERK antibody pulled down GlyRs α1^ins^ and β subunits (Fig 4C), while α3 was undetectable in ERK precipitates (Fig 4C). Glutathione S-Transferase (GST) fusion of IL of α1^ins^ (GST-α1^ins^-IL) also precipitated ERK from lysates of spinal cord slices (Fig 4D). By comparison, GST-fused IL of α1 (GST-α1-IL) did not interact with ERK (Fig 4D). DHPG (10 μM) treatment of slices for 3 min enabled GST-α1^ins^-IL to precipitate more ERK (Fig 4D). This increased binding was not due to altered ERK expression because there was no change of total ERK protein level after DHPG application (Fig 4D). Since DHPG increased ERK phosphorylation (Fig 4D), we examined whether α1^ins^ associated with the active ERK. The results showed that more phosphorylated ERK was pulled down by GST-α1^ins^-IL from DHPG-treated slices relative to control slices (Fig 4D).

To determine whether the phosphorylated ERK (*p*ERK) directly bound to α1^ins^, we purified His-tagged phosphorylated ERK2 (His-*p*ERK2) for in vitro GST pull-down assays [20]. GST-α1^ins^-IL exhibited a high affinity for His-*p*ERK2 (Fig 4E). Such an interaction was not observed when GST or GST-α1-IL was incubated with His-*p*ERK2 (Fig 4E). His-*p*ERK2 also failed to interact with GST-α3L-IL and GST-β-IL (Fig 4E), which harbored the ILs of the α3L and β subunits, respectively. By using nonphosphorylated His-ERK2, we found that none of the GlyR subunits bound to the inactive ERK2 (Fig 4F).

The α1^ins^ subunit differed from α1 only by the spliced insert of 8 amino acids in the IL. The specific α1^ins^/ERK interaction implicated that the spliced insert might be essential for ERK binding. Many proteins have been shown to interact with ERK through a consensus D-docking site (R/K-X_2-6_-Φ-X-Φ, in which Φ represents a hydrophobic amino acid) [21]. By analyzing the amino-acid sequence of α1^ins^, we found that the insertion of 8 amino acids (SPMLNLFQ) created a putative D-docking site. We therefore tested whether a synthetic peptide (FRRKRRHHKSPMLNLFQE), which encompassed the putative D-docking site, competed with α1^ins^ for ERK binding. This α1^ins^-derived peptide (pep-α1^ins^) was made cell permeable by addition of human immunodeficiency virus-type 1 TAT sequence (referred to as TAT-pep-α1^ins^). In the absence of TAT-pep-α1^ins^, GST-α1^ins^-IL effectively pulled down His-ERK2 from lysates of HEK293T cells coexpressing constitutively active MEK1(S218D/S222D) mutant (Fig 4G). Incubation with TAT-pep-α1^ins^ dose-dependently reduced the content of His-ERK2 pulled down by GST-α1^ins^-IL (Fig 4G). A TAT-fused scrambled peptide (referred to as TAT-Scram) had no effect even at the high dose of 10 μM (Fig 4G). These data suggested that the D-docking site of α1^ins^ mediated the binding to ERK. To consolidate this result, we performed coimmunoprecipitation ex vivo. Spinal DHPG (10 nmol, 10 min) application enhanced ERK interaction with α1^ins^ (Fig 4H). Pretreatment with TAT-pep-α1^ins^ (200 pmol) for 30 min disturbed α1^ins^/ERK interaction, whereas TAT-Scram had no effect (Fig 4H).

To test whether ERK binding resulted in α1^ins^ inhibition, we introduced TAT-pep-α1^ins^ (5 μM) into HEK293T cells that expressed α1^ins^ and mGluR5a. TAT-pep-α1^ins^ blocked DHPG from suppressing α1^ins^ currents (Fig 4I). In contrast, TAT-Scram (5 μM) did not affect DHPG-induced α1^ins^ inhibition (Fig 4I). In spinal slices, postsynaptic loading of TAT-pep-α1^ins^ also prevented DHPG from suppressing GlyR-IPSCs (Fig 4J). In the presence of TAT-Scram, however, a significant reduction of GlyRs-IPSCs was elicited by DHPG (Fig 4K). These data suggested that mGluR5 regulation of glycinergic currents relied on ERK/α1^ins^ interaction.

### ERK phosphorylated α1^ins^ at Ser380

**Fig 5:**
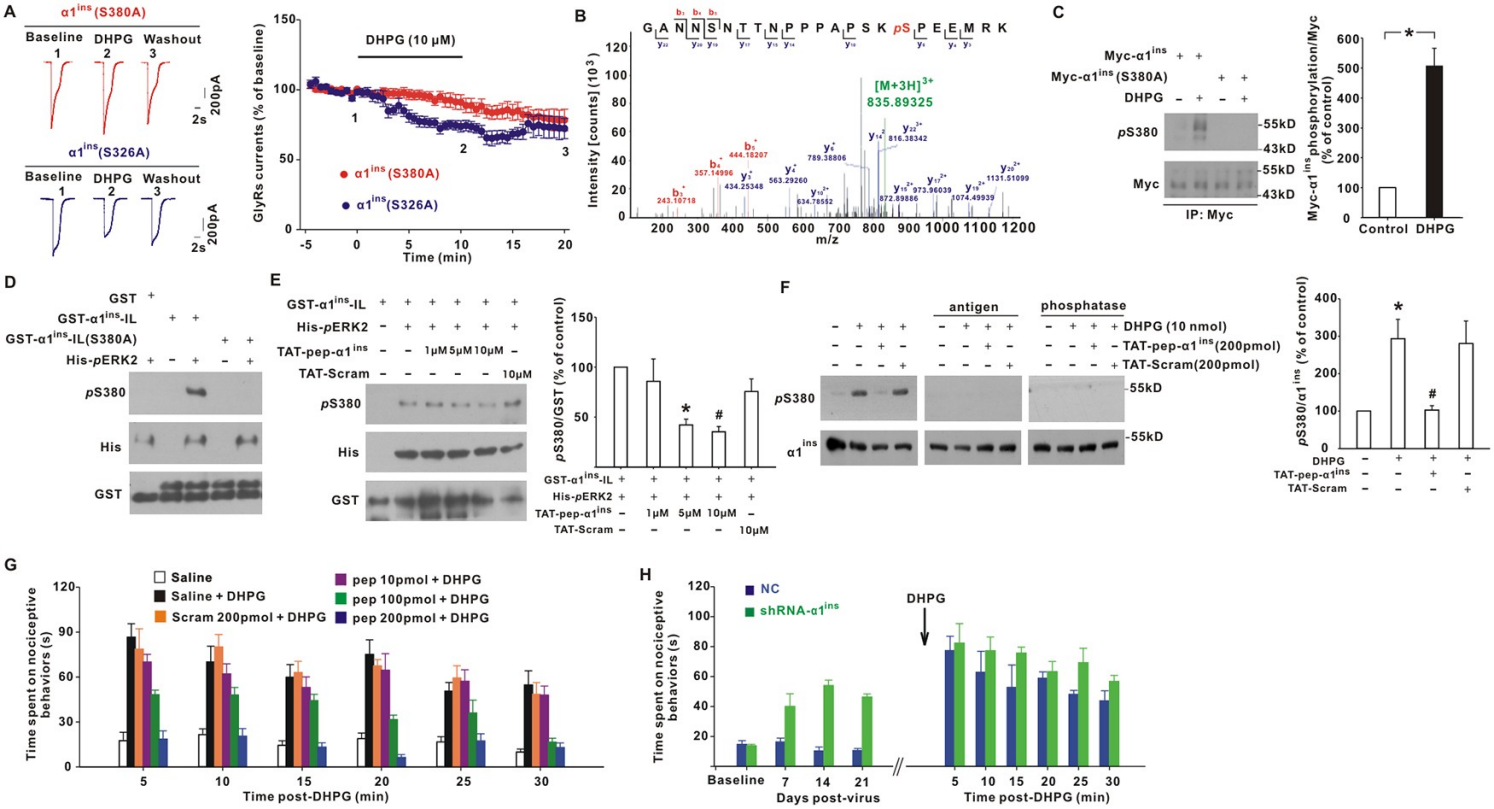
ERK phosphorylated α1^ins^ at Ser380. (**A**) DHPG reduced glycine (1 mM, 10 ms)-evoked currents in HEK293T cells expressing mGluR5a along with α1^ins^(S326A) (69.2 ± 3.5% of baseline at 10–15 min post-DHPG, *t*[8] = 3.978, *p* = 0.004, paired Student *t* test) but not with α1^ins^(S380A) (86.3 ± 5.1% of baseline at 10–15 min post-DHPG, *t*[8] = 1.974, *p* = 0.084). (**B**) LC MS/MS analysis identified Ser380 as a phosphorylation site on GST-α1^ins^-IL. (**C**) Myc-α1^ins^ or Myc-α1^ins^(S380A) was transfected along with mGluR5a in HEK293T cells, immunoprecipitated by Myc antibody, and immunoblotted with pS380-Ab. Representative western blots showed pS380-Ab signals with or without DHPG (10 μM) stimulation. *p = 0.002 (Mann–Whitney U test), *n* = 6. (**D**) Ser380 phosphorylation was catalyzed by ERK. GST-α1^ins^-IL or its mutant GST-α1^ins^-IL(S380A) was incubated in vitro with purified His-*p*ERK2 for 30 min before immunoblotting with pS380-Ab (up). The total His (middle) and GST proteins (bottom) were also probed. *n* = 6. (**E**) TAT-pep-α1^ins^ dose-dependently reduced GST-α1^ins^-IL phosphorylation catalyzed by His-*p*ERK2 in vitro. TAT-Scram was used as control. *p = 0.026, #*p* = 0.009 versus peptide-untreated group (one-way ANOVA with post hoc Bonferroni test), *n* = 6. (**F**) Intrathecal DHPG application (10 min) increased pS380-Ab signals, which was attenuated by pretreatment for 30 min with TAT-pep-α1^ins^ (left). Preabsorbing with excessive antigen (middle) or treatment with alkaline phosphatase (right) eliminated pS380-Ab signals. *p < 0.001 versus control, ^#^*p* < 0.001 versus DHPG (one-way ANOVA with post hoc Bonferroni test), *n* = 8. (**G**) Effects of intrathecal TAT-pep-α1^ins^ (pep) and TAT-Scram on DHPG (10 nmol)-induced spontaneous pain behaviors. F(25, 175) = 1.644, *p* = 0.033 (repeated measures ANOVA). *n* = 8 mice/group. (**H**) Intraspinal injection of AAV encoding shRNA-α1^ins^, but not NC, induced spontaneous pain behaviors (F[3, 30] = 15.459, *p* < 0.001, repeated measures ANOVA, *n* = 6 mice/group). Note that the painful behaviors in shRNA-α1^ins^ mice partially occluded those caused by subsequent DHPG application. The arrow indicated the time point when intrathecal DHPG was given at day 21 post-viral injection. The underlying data for this figure can be found in S1 Data. Error bars indicated SEM. AAV, adeno-associated virus; ANOVA, Analysis of Variance; DHPG, (S)-3,5-Dihydroxyphenylglycine; ERK, extracellular signal-regulated kinase; GlyR, glycine receptor; GST, Glutathione S-Transferase; HEK, human embryonic kidney; IL, intracellular large loop; LC MS/MS, liquid chromatograph/mass spectrometer; mGluR5, metabotropic glutamate receptor 5; NC, negative control shRNA; *p*ERK, phosphorylated ERK; pS380-Ab, phosphorylation-state–specific antibody against Ser380 on α1^ins^; shRNA, short hairpin RNA; TAT-pep-α1^ins^, TAT-fused α1^ins^-derived peptide; TAT-Scram, TAT-fused scrambled peptide.

Because ERK associated with and inhibited α1^ins^, we hypothesized that this kinase might act to phosphorylate α1^ins^. In the cytoplasmic region of α1^ins^, there are only two serine–proline motifs, ^380^SP and ^326^SP, which served as the potential phosphorylation sites by proline-directed ERK kinase. To investigate whether these two serine residues regulated α1^ins^ function, we constructed α1^ins^(S380A) and α1^ins^(S326A) mutants in which Ser380 and Ser326 were substituted with alanine, respectively. In HEK293T cells, these mutants responded to exogenously applied glycine with large membrane currents (Fig 5A). DHPG stimulation of coexpressed mGluR5a reduced the peak amplitudes of α1^ins^(S326A) (Fig 5A). In α1^ins^(S380A)-expressing cells, however, the inhibitory effect of DHPG was blocked (Fig 5A), suggesting the importance of Ser380 in the regulation of α1^ins^ currents.

To identify the exact phosphorylation site on α1^ins^, we incubated GST-α1^ins^-IL for 30 min with lysates of HEK293T cells coexpressing MEK1(S218D/S222D) and His-ERK2 [20,22]. The GST protein was then isolated for mass spectrometry. This approach identified Ser380 as a novel, to our knowledge, phosphorylation site (Fig 5B). We raised a rabbit phosphorylation-state–specific antibody against Ser380 on α1^ins^ (referred to as pS380-Ab). When Myc-tagged α1^ins^ was immunoprecipitated from transfected HEK293T cells, the pS380-Ab detected a weak phosphorylation signal (Fig 5C). DHPG activation of co-transfected mGluR5a significantly enhanced the level of Myc-α1^ins^ phosphorylation (Fig 5C). When Ser380 was mutated to alanine, the pS380-Ab signal was totally abolished (Fig 5C).

To test whether ERK was the kinase that catalyzed Ser380 phosphorylation, we conducted the kinase assays in vitro. In the absence of active ERK, pS380-Ab did not detect any phosphorylation signal on GST-α1^ins^-IL (Fig 5D). Incubation with purified His-*p*ERK2 noticeably elevated the phosphorylation level of GST-α1^ins^-IL (Fig 5D). This phosphorylation was eliminated by Ser380 mutation to alanine (Fig 5D). The addition of TAT-pep-α1^ins^ in the reaction buffer also inhibited GST-α1^ins^-IL phosphorylation in a dose-dependent manner (Fig 5E), implying that Ser380 was the amino-acid residue that was directly phosphorylated by ERK. We then examined α1^ins^ phosphorylation in spinal dorsal horn. The protein band recognized by pS380-Ab ex vivo predominantly migrated at 55 kDa (Fig 5F). To confirm the specificity of pS380-Ab, we incubated the antibody with excess immunizing antigen before western blot or treating the immunoblots with alkaline phosphatase, finding that both of them erased pS380 signals (Fig 5F). Intrathecal application of DHPG enhanced pS380-Ab signals (Fig 5F) and meanwhile evoked spontaneous nociceptive behaviors (Fig 5G). Pretreatment with TAT-pep-α1^ins^ not only inhibited Ser380 phosphorylation induced by DHPG (Fig 5F) but also alleviated the nociceptive behaviors (Fig 5G). We found that knockdown of α1^ins^ by intraspinal injection of AAV encoding shRNA-α1^ins^ mimicked DHPG by eliciting spontaneous pain behaviors (Fig 5H). It was noteworthy that intrathecal application of DHPG at day 21 post-viral injection enhanced the nociceptive behaviors to a lesser degree in shRNA-α1^ins^ mice than in NC-injected mice (Fig 5H). The maximum nociceptive responses observed at 5 min post-DHPG were only 183.2 ± 35.2% of pre-DHPG values in shRNA-α1^ins^ mice compared to 915.5 ± 301.2% in NC mice (*p* = 0.002 relative to shRNA-α1^ins^ mice, Mann–Whitney U test, *n* = 6 mice/group), suggesting that the painful responses elicited by shRNA-α1^ins^ partially occluded DHPG action. These results confirmed that down-regulation of α1^ins^ function was one of the important ways for mGluR5 to sensitize the nociceptive behaviors.

### Ser380 phosphorylation induced the ubiquitination and endocytosis of α1^ins^

**Fig 6:**
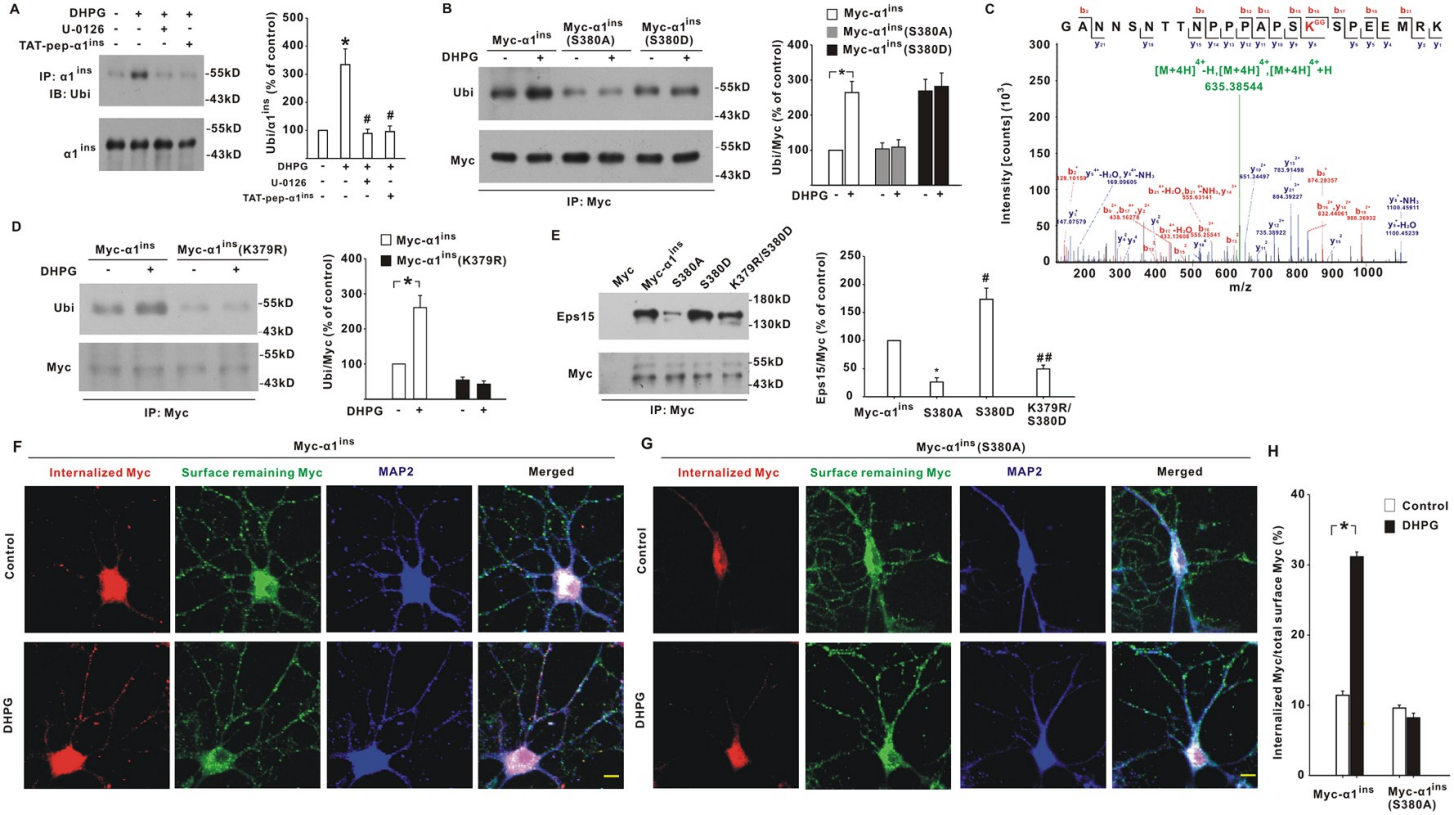
Ser380 phosphorylation promoted α1^ins^ ubiquitination and endocytosis. (**A**) Intrathecal application of DHPG (10 nmol, 10 min) enhanced α1^ins^ ubiquitination. Effects of spinal U-0126 (2 nmol) and TAT-pep-α1^ins^ (200 pmol) were also examined. *p < 0.001 versus control, #*p* < 0.001 versus DHPG (one-way ANOVA with post hoc Bonferroni test), *n* = 6. (**B**) Myc-α1^ins^(S380A) inhibited, while Myc-α1^ins^(S380D) occluded, the increase of Myc-α1^ins^ ubiquitination caused by DHPG in cultured neurons. *p = 0.004 versus control (one-way ANOVA with post hoc Bonferroni test), *n* = 6. (**C**) LC MS/MS analysis identified Lys379 on Myc-α1^ins^ as a ubiquitination site. (**D**) DHPG-induced Myc-α1^ins^ ubiquitination was attenuated by Lys379 mutation to Arginine. *p < 0.001 versus DHPG-untreated control (one-way ANOVA with post hoc Bonferroni test), *n* = 6. (**E**) Eps15 contents precipitated by Myc antibody from neurons transfected with Myc, Myc-α1^ins^, Myc-α1^ins^(S380A), Myc-α1^ins^(S380D), or Myc-α1^ins^(K379R/S380D). *p = 0.001 and ^#^*p* = 0.001 versus Myc-α1^ins^, ^##^*p* < 0.001 versus Myc-α1^ins^(S380D) (one-way ANOVA with post hoc Bonferroni test), *n* = 6. (**F–H**) Internalized (red) and surface remaining Myc-α1^ins^ (green, **F**) or Myc-α1^ins^(S380A) (green, **G**) in cultured neurons untreated or treated with DHPG (10 μM). The ratios of internalized to total fluorescence intensities were averaged (**H**). *p < 0.001 versus DHPG-untreated control (Mann–Whitney U test), *n* = 30 cells/group. Scale bar, 5 μm. The underlying data for this figure can be found in S1 Data. Error bars indicated SEM. ANOVA, Analysis of Variance; DHPG, (S)-3,5-Dihydroxyphenylglycine; Eps15, epidermal growth factor receptor substrate 15; IP, immunoprecipitation; LC MS/MS, liquid chromatograph/mass spectrometer; MAP2, Microtubule-Associated Protein 2; TAT-pep-α1^ins^, TAT-fused α1^ins^-derived peptide; Ubi, ubiquitin.

As mentioned above, the polypeptide detected by pS380-Ab migrated at 55 kDa in the spinal dorsal horn compared to 48 kDa of intact α1^ins^ (Fig 5F). Previous studies have indicated that GlyR α1 subunit can be ubiquitinated when expressed in *Xenopus* oocytes [13], which causes about a 7-kDa upward mobility shift. Our data showed that spinal α1^ins^ was also a ubiquitinated protein, with the apparent molecular size of 55 kDa (Fig 6A). Ubiquitin (Ubi) modification of α1^ins^ was activity-dependent. Stimulation of mGluR5 significantly enhanced α1^ins^ ubiquitination level (Fig 6A). When ERK was inhibited by U-0126, DHPG-induced α1^ins^ ubiquitination was substantially repressed (Fig 6A). A similar inhibition was also observed when TAT-pep-α1^ins^ was used to disturb ERK/α1^ins^ interaction (Fig 6A). These data raised the possibility that Ser380 phosphorylation might favor α1^ins^ ubiquitination. To test this, we expressed Myc-α1^ins^, Myc-α1^ins^(S380A), or Myc-α1^ins^(S380D) in neurons along with GlyR β subunit. The nonphosphorylatable Myc-α1^ins^(S380A) inhibited, whereas phospho-mimicking Myc-α1^ins^(S380D) occluded, the stimulatory effect of DHPG on Myc-α1^ins^ ubiquitination (Fig 6B), suggesting that Ser380 phosphorylation facilitated the conjugation of Ubi to α1^ins^.

There are 10 potential ubiquitination sites (lysine residues) within the IL of α1^ins^. We used DHPG to treat neurons expressing Myc-α1^ins^/β subunits and immunoprecipitated Myc protein for mass spectrometry. This method identified Lys379 as a ubiquitinated site on Myc-α1^ins^ (Fig 6C). Mutation of Lys379 to arginine attenuated the basal ubiquitination of Myc-α1^ins^ (Fig 6D) and blocked DHPG from enhancing α1^ins^ ubiquitination level (Fig 6D). Lys379 mutation did not eliminate the Ubi signal completely (Fig 6D), suggesting that Lys379 was the major but not the sole site for Ubi modification.

The ubiquitinated cargos on plasma membrane can be recognized by endocytic machinery, which is a critical step for the initiation of endocytic process. By performing coimmunoprecipitation in cultured neurons, we found a physical interaction of Myc-α1^ins^ with epidermal growth factor receptor substrate 15 (Eps15) (Fig 6E), one of the key endocytic components that recruit the ubiquitinated proteins [23]. Importantly, the molecular interaction of Myc-α1^ins^ with Eps15 was regulated by Ser380 phosphorylation. Compared to Myc-α1^ins^, Myc-α1^ins^(S380A) pulled down less Eps15 (Fig 6E). In contrast, the Eps15 contents precipitated by Myc-α1^ins^(S380D) were higher than those by Myc-α1^ins^ (Fig 6E). When Lys379 was mutated to arginine, the interaction between Myc-α1^ins^(S380D) and Eps15 was attenuated (Fig 6E). Immunocytochemical analysis showed that shRNA knockdown of Eps15 (S6A Fig) prevented the decrease of surface Myc-α1^ins^ expression induced by DHPG (S6B Fig). To directly examine whether Ser380 phosphorylation led to α1^ins^ endocytosis, we transfected Myc-α1^ins^ or Myc-α1^ins^(S380A) along with GlyR β subunit in neurons. DHPG induced a marked increase of internalized Myc-α1^ins^ immunoreactivity when compared to media control (Fig 6F and 6H). There was no difference in the immunofluorescence intensities of internalized Myc-α1^ins^(S380A) between control and DHPG-treated cells (Fig 6G and 6H). These data suggested that mGluR5 caused α1^ins^ endocytosis through Ser380 phosphorylation.

### Potentiation of glycinergic neurotransmission by TAT-pep-α1^ins^ alleviated pathological pain

**Fig 7:**
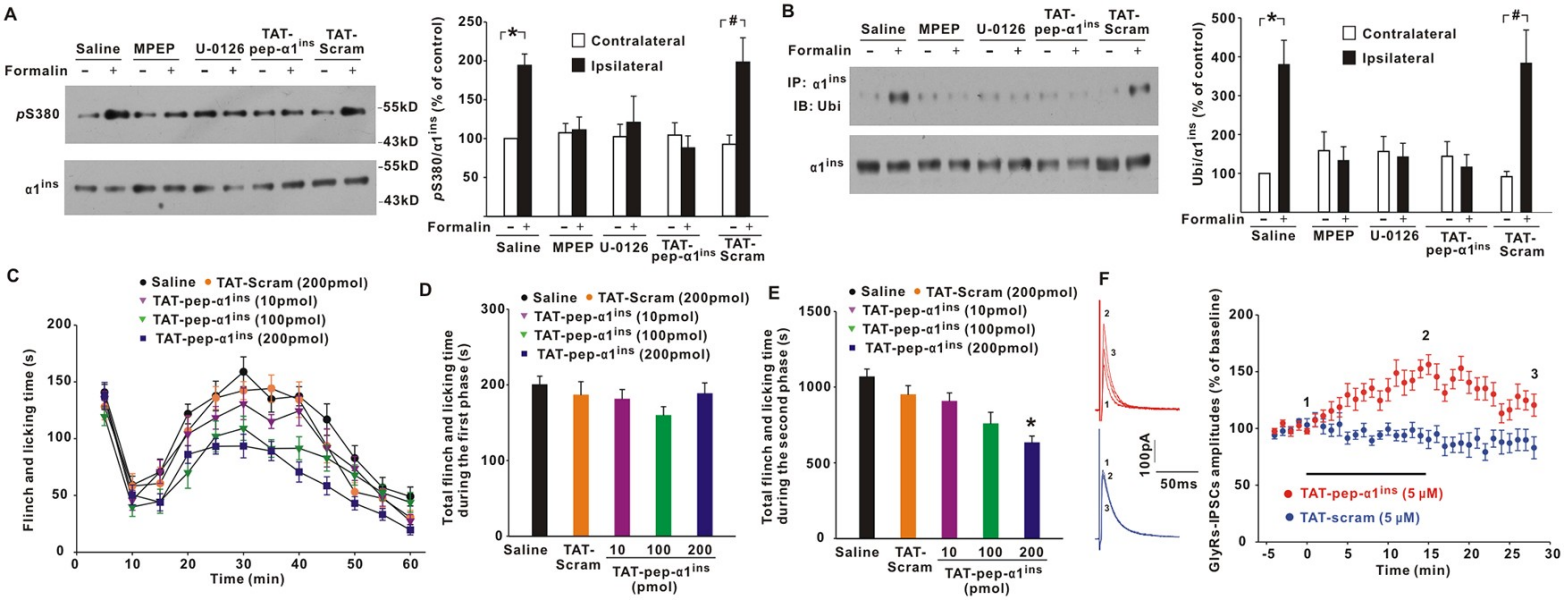
TAT-pep-α1^ins^ attenuated inflammatory pain. (**A**) Formalin injection into left hindpaws of mice enhanced *p*S380 in the ipsilateral dorsal horn of spinal cord. Effects of intrathecal MPEP (50 nmol), U-0126 (2 nmol), TAT-pep-α1^ins^, or TAT-Scram (200 pmol) on *p*S380 were also examined. *p = 0.017, #*p* = 0.007 versus contralateral sides (one-way ANOVA with post hoc Bonferroni test), *n* = 10. (**B**) Effects of formalin on α1^ins^ ubiquitination. *p = 0.002, #*p* = 0.002 versus contralateral sides (one-way ANOVA with post hoc Bonferroni test), *n* = 6. (**C**) Effects of TAT-pep-α1^ins^ and TAT-Scram on formalin-induced spontaneous pain. F(44, 396) = 1.652, *p* = 0.007 (repeated measures ANOVA). *n* = 10 mice/group. (**D–E**) The first-phase (0–10 min, **D**) and second-phase (15–60 min, **E**) behaviors in formalin tests were summarized. *p = 0.004 versus TAT-Scram–treated mice (one-way ANOVA with post hoc Bonferroni test). (**F**) GlyR-IPSCs in slices from formalin-injected mice were potentiated by TAT-pep-α1^ins^ (144.9 ± 7.6% of baseline at 15–20 min postpeptide, *t*[15] = 6.495, *p* < 0.001, paired Student *t* test) but not by TAT-Scram (88.0 ± 6.5% of baseline at 15–20 min postpeptide, *t*[10] = 1.856, *p* = 0.093). The underlying data for this figure can be found in S1 Data. Error bars indicated SEM. ANOVA, Analysis of Variance; GlyR, glycine receptor; IP, immunoprecipitation; IPSC, inhibitory postsynaptic current; MPEP, 6-Methyl-2-(phenylethynyl) pyridine; *p*S380, phosphorylation at Ser380; TAT-pep-α1^ins^, TAT-fused α1^ins^-derived peptide; TAT-Scram, TAT-fused scrambled peptide; Ubi, ubiquitin.

Glycinergic disinhibition following peripheral injury plays an important role in central sensitization of nociceptive behaviors. To examine the role of α1^ins^ in inflammatory pain, we injected formalin into left hindpaws of mice. Our data showed that Ser380 phosphorylation was significantly enhanced in the dorsal horns ipsilateral to formalin injection relative to contralateral sides (Fig 7A). Coincident with Ser380 phosphorylation was a robust α1^ins^ ubiquitination on the injured sides (Fig 7B). The α1^ins^ phosphorylation (Fig 7A) and ubiquitination (Fig 7B) were inhibited when mGluR5 inhibitor MPEP (50 nmol) or ERK inhibitor U-0126 (2 nmol) was intrathecally applied for 30 min before formalin injection. Disruption of ERK/α1^ins^ interaction by TAT-pep-α1^ins^ (200 pmol) also blunted α1^ins^ phosphorylation (Fig 7A) and ubiquitination (Fig 7B) in formalin mice. The same dose of TAT-Scram, however, had no effect (Fig 7A and 7B). Formalin triggers biphasic spontaneous pain behaviors (Fig 7C). We found that TAT-pep-α1^ins^ dose-dependently ameliorated the second-phase painful behaviors (Fig 7C and 7E). Compared to saline control, TAT-Scram had no effect on the second-phase responses (Fig 7C and 7E). The first phase was similar among saline-, TAT-Scram–, and TAT-pep-α1^ins^–treated mice (Fig 7C and 7D). These data suggested that mGluR5/ERK-signaling–dependent α1^ins^ phosphorylation and ubiquitination closely correlated with inflammatory pain. We also tested the effect of TAT-pep-α1^ins^ (200 pmol) on the neuropathic pain induced by spared nerve injury. The results illustrated that interference with ERK/α1^ins^ interaction alleviated the mechanical allodynia (S7 Fig). As a control, TAT-Scram (200 pmol) produced no significant effect on the pathological pain (S7 Fig).

To test whether disruption of ERK/α1^ins^ interaction potentiated glycinergic transmission in inflamed mice, we recorded GlyR-IPSCs in spinal slices prepared at 90 min post-formalin injection. The results showed that bath application of TAT-pep-α1^ins^, but not TAT-Scram, enhanced the amplitudes of GlyR-IPSCs (Fig 7F). If α1^ins^ was virally knocked down before formalin injection, the synaptic potentiation by TAT-pep-α1^ins^ was eliminated (S8A Fig). In α3-deleted inflamed mice, however, TAT-pep-α1^ins^ still boosted GlyR-IPSCs (S8B Fig). TAT-pep-α1^ins^ did not regulate the synaptic responses mediated by α-Amino-3-hydroxy-5-methylisoxazole-4-propionic Acid (AMPA) receptors (S8C Fig), N-methyl-D-aspartate (NMDA) receptors (S8D Fig), or GABA_A_ receptors in inflamed mice (S8E Fig). In intact mice, glycinergic transmission was also insensitive to TAT-pep-α1^ins^ (S8F Fig), possibly because of low ERK activity at resting conditions. These data suggested that specific reinstatement of α1^ins^-mediated glycinergic transmission attenuated inflammatory pain.

## Discussion

GlyR α1 subunit is abundantly expressed in the adult spinal cord and brain stem and is responsible for the majority of glycinergic neurotransmission [2,5]. The importance of α1 in neuronal function is underlined by genetic studies showing that mutation or reduced expression of α1 in the spinal cord and brain stem incurs severe neurological disorders such as hyperekplexia [24–26]. The major finding in the current study was that α1^ins^, a longer α1 variant, was involved in spinal glycinergic transmission. The α1^ins^ expression was restricted to the superficial dorsal horn of spinal cord, and knockdown of α1^ins^ did not affect motor coordination but elicited pain hypersensitivity, suggesting that α1^ins^ was required for spinal nociceptive processing. Activation of mGluR5 in the spinal cord dorsal horn has been shown to enhance nociceptive neuronal excitability, potentiate glutamatergic inputs, and play an important role in pathological pain [27–31]. We provided evidence that mGluR5 activation also attenuated glycinergic currents through ERK-dependent α1^ins^ phosphorylation. This novel, to our knowledge, signaling pathway might act synergistically with the enhanced glutamatergic transmission and neuronal excitability to sensitize the nociceptive behaviors. In support of this notion, α1^ins^ knockdown partially occluded DHPG action in inducing painful responses, and, more importantly, specific recovery of α1^ins^-mediated glycinergic inhibition attenuated pathological pain.

The IL between TM3–TM4 of GlyRs subunits is a unique domain that displays the highest degree of variability. These loops bind to intracellular scaffolds and signaling components that are essential for the activity-dependent modification of glycinergic efficacy. Great efforts have been made to distinguish the biological characteristics of two GlyR α3 subunits, α3K and the longer α3L that contains additional 15 amino acids in the TM3–TM4 loop [32]. These two α3 variants are distributed throughout the central nervous system. However, they differ significantly in terms of channel desensitization, membrane distribution, and synaptic localization [33–36]. The alternatively spliced insert in α3L has been proposed to stabilize the secondary structure of the TM3–TM4 loop and regulate the channel gating [34]. The current study demonstrated that one of the important functions of the α1^ins^ insert was to constitute a D-docking site, a unique structure that allowed mGluR5/ERK signaling to decrease α1^ins^-mediated glycinergic inhibition and evoke nociceptive behavioral sensitization. The deficiency of the spliced insert and corresponding D-docking site explained why α1 subunit was refractory to mGluR5 regulation. As with mGluR5, the adenosine A1 receptor has also been shown to regulate glycinergic responses mediated by α1^ins^ but not by α1 [15]. These data implied that α1 and α1^ins^ might respond distinctly to some G-protein–coupled receptors despite their high similarities in electrophysiological and pharmacological properties [6].

Phosphorylation plays an important role in the dynamic modulation of ligand-gated ion channels. Most of the known phosphorylation sites on GlyRs have been mapped to the TM3–TM4 loop. The cyclic adenosine monophosphate (cAMP)-dependent protein kinase (PKA) can suppress glycinergic transmission by phosphorylating α3L at Ser346, which is involved in the regulation of nociceptive behaviors and respiratory rhythm [19,37]. Ser346 phosphorylation also enables allosteric modulators to interact with α3L and reverse inflammation-induced glycinergic disinhibition in the spinal cord [38]. PKC phosphorylation of GlyR β subunit disturbs the binding of the postsynaptic scaffold protein gephyrin and decreases GlyR accumulation at inhibitory synapses [11]. mGluR5 has been well known to regulate a wide range of cellular responses through PKC pathway. We tested the role of PKC in mGluR5 modification of GlyRs, finding that glycinergic responses were insensitive, at least in part, to PKC signaling initiated specifically by mGluR5. Previous studies have demonstrated that mGluR5 can stimulate a transient activation of ERK through protein phosphatase 2A and Homer1b/c pathways [16,17]. Our data revealed that ERK was essential for mGluR5 to suppress glycinergic currents. Upon binding to the D-docking site of α1^ins^, ERK catalyzed the phosphorylation of Ser380 and led to the endocytosis of α1^ins^. The activity-dependent Ser380 phosphorylation of α1^ins^ thus constituted a novel, to our knowledge, mechanism to modulate the efficacy of glycinergic transmission. We found that Ser380 phosphorylation was closely associated with inflammatory pain. Interference with ERK/α1^ins^ interaction decreased Ser380 phosphorylation in inflamed mice, resumed glycinergic transmission, and attenuated pain sensitization. The spliced insert also introduced a serine–proline motif (^326^SP) in the TM3–TM4 loop of α1^ins^. However, this SP motif was found to be dispensable for mGluR5 inhibition of α1^ins^ currents.

Our data showed that α1^ins^ was a ubiquitinated protein, and Lys379 was identified as the major ubiquitination site. The ubiquitination decreased the band mobility of α1^ins^ by about 7 kDa, suggesting that only one Ubi molecule was conjugated to one α1^ins^ subunit. Monoubiquitination generally regulates protein–protein interaction and the endocytic process [39]. The Ubi moieties on α1^ins^ were recognized by endocytic adaptor protein Eps15, which initiated α1^ins^ endocytosis to remove glycinergic inhibition [39]. Importantly, activation of mGluR5/ERK signaling or Ser380 mutation to aspartic acid enhanced the ubiquitination level of α1^ins^, indicating that α1^ins^ ubiquitination was regulated by Ser380 phosphorylation. Possibly, Ser380 phosphorylation facilitated the interaction of α1^ins^ with unidentified ubiquitination machinery that catalyzed the Ubi transfer cascade. Alternatively, Ser380 phosphorylation caused the conformational change in the intracellular large loop of α1^ins^ so that lysine residues became susceptible for Ubi conjugation reaction. Recently, HECT, UBA, WWE domain containing 1 (HUWE1) is identified as the E3 Ubi ligase that contributes to the ubiquitination and endocytosis of spinal GlyR α1 subunit during inflammatory pain [40]. HUWE1 knockdown enhances glycinergic transmission and generates an effective analgesic action against pain hypersensitivity [40]. The E3 Ubi ligases that ubiquitinate α1^ins^ subunit require further investigation.

Taken together, the current study demonstrated that GlyR α1^ins^ subunit served as a specific target for mGluR5/ERK signaling to reduce glycinergic inhibition and evoke spinal sensitization. Given the gating control by glycinergic inhibition over nociceptive sensory input through the spinal cord dorsal horn to higher brain regions, these data shed new light on a potential for α1^ins^ to treat pathological pain.

## Materials and methods

### Ethics statement

The animal experiments were conducted in accordance with the guidelines of the Animal Care and Use Committee of Lanzhou University. The male C57BL/6J mice (10–12 weeks old) were purchased from the Experimental Animal Center of Lanzhou University (approval number: SCXK(GAN)-2013-0002) and selected randomly in each experiment. These animals were housed two to three per cage with free access to food and water on a 12 h light/dark cycle. Every effort was made to minimize the number and suffering of animals.

### Expression constructs

The cDNAs encoding full-length human or mouse α1, α1^ins^, α3L, and β subunits were subcloned into pcDNA3.1 vector and obtained from Youbio Biotechnologies (Changsha, China). The site-directed mutagenesis was used to generate α1^ins^(S380A), α1^ins^(S380D), α1^ins^(S326A), α1^ins^(K379R), and α1^ins^(K379R/S380D) (the number did not include the signal peptide sequence). The isolated α1^ins^ or its mutant cDNAs were modified by inserting a c-Myc sequence (EQKLISEEDL) between the second and third amino acids. The IL of α1^ins^ (residues 308–400), α1 (residues 308–392), β (residues 329–456), and α3L (residues 308–400) were PCR subcloned and ligated into pGEX-6p-1 vector. Human GRM5 in Tango vector was a gift from Bryan Roth (Addgene plasmid #66390; Watertown, MA, USA). The rat ERK2 cDNA in pcDNA3.1-myc-HisA were obtained from Genewiz Inc. (Suzhou, China). The pBabe-puro vector encoding MEK1(S218D/S222D) was a gift from William Hahn (Addgene plasmid #15268). All constructs were verified by DNA sequencing. The shRNA sequences targeting mouse α1^ins^, α3, and Eps15 were 5′-GCCCCATGCTAAATCTGTTTC-3′, 5′-GCCAAAGAGCCCTGATGAAAT-3′, and 5′-GCTTCCAGACTTGATTCTTGG-3′, respectively. The NC sequence was 5′-TTCTCCGAACGTGTCACGT-3′. These shRNAs were inserted into pGPU6-GFP vector and commercially obtained from GenePharma (Shanghai, China). The AAV (AAV-DJ; 6 × 10^9^ TU/ml) obtained from GenePharma was used to deliver GFP and shRNA.

### Reagents

TAT-fused 18-amino–acid peptide (FRRKRRHHKSPMLNLFQE) and its scrambled control peptide (LERPHMRQRFHNRFKSLK) were obtained from Synpeptide (Shanghai, China). DHPG, GDP-β-S, and dynasore (Sigma-Aldrich, St. Louis, MO, USA) were dissolved in the internal solution or artificial cerebrospinal fluid (ACSF, in mM: 119.0 NaCl, 2.5 KCl, 2.5 CaCl_2_, 1.3 MgCl_2_, 1.0 NaH_2_PO_4_, 26.0 NaHCO_3_, 11.0 D-glucose [pH 7.4]). CHPG, MPEP, CPCCOEt, U-0126, chelerythrine, PD98059, Ro-32-0432 (Sigma-Aldrich), and AM251 (Absin, Shanghai, China) were dissolved in dimethyl sulfoxide, which was diluted with ACSF or internal solution just before use. The final concentration of dimethyl sulfoxide was less than 0.1%.

### Animal models and drug delivery

Formalin (1.5%, 20 μl) was injected subcutaneously into the plantar surfaces of hindpaws. For spared nerve injury [41], the mouse was anaesthetized with sodium pentobarbital (60–90 mg/kg, i.p.), and the skin of the left lateral thigh was incised to expose the sciatic nerve. After careful separation of the three nerve branches, the tibial and common peroneal nerves were ligated with 5.0 silk and transected, followed by removing a 2–3 mm portion of the tibial and common peroneal nerves distal of the ligation. Every effort was made to keep the sural nerve intact during the operation. The muscle and skin were then closed in layers. Intrathecal injection (5 μl) was achieved by direct lumbar puncture as described previously [20]. Intraspinal viral injection was conducted in sodium pentobarbital (60–90 mg/kg, i.p.)-anesthetized mice [42]. Briefly, the animals were immobilized on a stereotaxic frame after a laminectomy. A glass pipette attached to a 5-μl microsyringe was used to inject the viral vectors (30 nl/min) at a depth of 0.2–0.3 mm from the dorsal surface of lumbar segment and 0.5 mm apart from the midline. After the injection, the muscle and skin were closed.

### Cell cultures and transfection

The HEK293T cells were plated onto poly-D-lysine (0.1 mg/ml)-coated coverslips, maintained in Dulbecco’s modified Eagle’s medium (DMEM) supplemented with 10% fetal bovine serum and 1% penicillin/streptomycin, and grown at 37 °C. The spinal cord neurons were cultured as previously described [43]. In brief, the mouse pups (postnatal day 1) were decapitated, and the spinal cords were removed into ice-cold Hank’s Balanced Salt Solution containing 10 mM HEPES. After careful removal of all meninges, the dorsal quadrants of spinal cords were dissected out, chopped into small strips, and digested by papain (2 mg/ml) for 20–30 min at 37 °C. DMEM with 10% heat-inactivated fetal bovine serum was added to terminate the digestion reaction. After trituration, the cells were harvested by centrifugation at 1,000 × *g* and resuspended in neurobasal medium containing 2% fetal bovine serum, 2% heat-inactivated horse serum, 2% B27, 1% penicillin/streptomycin, and 2 mM L-glutamine. The neurons were plated onto poly-D-lysine–coated coverslips with the cell density adjusted to be 1.5 × 10^6^. The cultured cells were transfected with Lipo^6000^ Transfection Reagent (Beyotime Institute of Biotechnology, Jiangsu, China) according to the manufacturer’s instructions.

### GST pull-down assay

GST-fused proteins were expressed in *Escherichia coli* BL21 cells and affinity purified with glutathione agarose beads (Sigma-Aldrich) [20]. His6-tagged recombinant proteins were purified with nickel-nitrilotriacetic acid column (Roche, Indianapolis, IN, USA) from lysates of transfected HEK293T cells and eluted by 0.25 M imidazole, 300 mM NaCl, and 50 mM NaH_2_PO_4_ (pH 8.0) [20]. Amicon Ultra Centrifugal Filters (Millipore, Burlington, MA, USA) were used to concentrate and desalt the eluted His proteins. The His-tagged phosphorylated ERK2 was purified from lysates of HEK293T cells coexpressing MEK1(S218D/S222D) and His-ERK2 [20]. The purity of His protein was assessed by western blot and Coomassie blue staining. For GST pull-down, the purified His proteins (0.5 μM) or lysates (200 μg) of spinal dorsal horn or HEK293T cells were incubated with glutathione-agarose-bead–bound GST proteins in radio-immunoprecipitation assay (RIPA) buffer (50 mM Tris·HCl [pH 8.0], 150 mM NaCl, 1 mM EDTA, 1.0% NP-40, 0.1% SDS, 0.5% sodium deoxycholate, and inhibitor cocktail of phosphatases and proteases). After gentle rotation for 4 h at 4 °C, the beads were collected by brief centrifugation at 800 × *g*, washed for six times with RIPA buffer, and boiled in SDS sample buffer. Different concentrations of peptides were preincubated with His proteins for 1 h before pull-down assays.

### Immunoprecipitation and western blot

The mice were anesthetized with sodium pentobarbital and decapitated. The L4–L5 spinal cord was removed into ice-cold ACSF bubbled with 95% O_2_ and 5% CO_2_. The dorsal quadrant of L4–L5 spinal cord was dissected out and homogenized in ice-cold RIPA buffer. HEK293T cells and cultured neurons were lysed in RIPA buffer. After centrifugation at 14,000 × *g* for 10 min, the supernatants were collected and incubated at 4 °C with the indicated primary antibody overnight. The protein A/G-Agarose beads were incubated with the immune complexes for 4 h. After extensive washes, the immunoprecipitates were resuspended in SDS sample buffer and boiled for 5 min. The protein samples were subjected to SDS-Polyacrylamide Gel Electrophoresis (SDS-PAGE) and transferred to polyvinylidene difluoride membranes. After blocking with 5% nonfat milk, the membranes were incubated with primary antibodies overnight at 4 °C, followed by incubation with horseradish-peroxidase–conjugated secondary antibody (Jackson ImmunoResearch Laboratories, Baltimore, PA, USA). The blots were visualized by enhanced chemiluminescence. The primary antibodies used in the present study included rabbit anti-GlyR-α1 antibody from Synaptic System (#146003, Gottingen, Germany) or Proteintech (#17951-1-AP, Rosemont, IL, USA); rabbit anti-Myc antibody from Abcam (#AB9106; Cambridge, UK); mouse anti-Myc antibody from Santa Cruz Biotechnology (#sc-40; Santa Cruz, CA, USA); mouse anti-Ubi antibody from Sigma-Aldrich (#U0508); mouse anti-His (#E0021) and mouse anti-GST antibody (#E0019) from Anbo Biotechnology (JiangSu, China); rabbit anti-GlyR-α3 (#13145), rabbit anti-GlyR-β (#15371), and mouse anti-ERK1/2 antibody (#66192) from Proteintech; mouse anti-Eps15 antibody from BD Transduction Laboratories (#610806; Franklin Lakes, NJ, USA); rabbit anti-ERK1/2 (#9102) and mouse anti-ERK1/2-pThr183/Tyr185 antibody (#9106) from Cell Signaling (Beverly, MA, USA); and mouse anti-β-actin antibody (#A5316) from Sigma-Aldrich. The phosphopeptide Cys-PSK*p*SPEEMR was synthesized to raise rabbit anti-pSer380 antibody (Proteintech), and Cys-NLFQDDEGGEGRFN was used to raise rabbit anti-α1^ins^ antibody (Genscript, Nanjing, China).

### Kinase assay in vitro

The *p*ERK2 (0.5 μM) was incubated with glutathione-agarose-bead–bound GST-α1^ins^-IL or GST-α1^ins^-IL(S380A) in 50-μl phosphorylation buffer (50 mM morpholinepropanesulfonic acid [MOPS] [pH 6.5], 100 μM ATP, 10 mM MgCl_2_, 1 mM EGTA) [44]. After 30-min reaction at 30 °C, the beads were collected by centrifugation at 1,000 × *g* and washed extensively with RIPA. The peptides at different concentrations were included in the reaction buffer when indicated. For phosphorylation assay with cell lysates as the source of kinases [22], HEK293T cells expressing MEK1(S218D/S222D) and His-ERK2 were lysed in hypoosmotic solution (50 mM MOPS [pH 6.5], 1 mM EGTA, and phosphatase and protease inhibitors). After centrifugation at 14,000 × *g* for 5 min, the supernatant was collected and supplemented with 50 mM MOPS (pH 6.5), 100 μM ATP, 10 mM MgCl_2_, 1 mM EGTA, 1 mM dithiothreitol, and phosphatase/protease inhibitors. GST proteins were incubated with the cell lysates for 30 min at 30 °C.

### LC MS/MS analysis

GST proteins purified by glutathione agarose beads or the immunoprecipitated Myc-α1^ins^ from neurons were separated by SDS-PAGE. The corresponding protein band was then excised and cut into 1-mm pieces. After in-gel digestion with trypsin (10 ng/μl) at 37 °C overnight, the peptides were extracted with 50% acetonitrile/0.1% trifluoroacetic acid and dried. The tryptic peptides were dissolved in 0.1% formic acid (solvent A); loaded onto a 5-cm–long, 75-μm-inner–diameter trap column packed with 5-μm C18 stationary phase; and separated by 15-cm–long, 75-μm-inner–diameter analytical column packed with 2-μm C18 stationary phase. The gradient was comprised of 5%–35% solvent B (0.1% formic acid in 80% acetonitrile) for 60 min, 35%–80% solvent B for 20 min, and 100% solvent B for 10 min at a constant flow rate of 300 nl/min on an EASY-nLC 1200 UPLC system (Thermo Fisher Scientific, Waltham, MA, USA). The eluted peptides were subjected to Thermo Scientific Obritrap Fusion Lumos Tribrid mass spectrometer. The electrospray voltage was 2.5 kV. The mass spectrometer was operated in the data-dependent mode, with a survey scan over an m/z range of 300–1,800 at a resolution of 120,000 in the Orbitrap. Data were processed using the Proteome Discoverer 2.1 software package (Thermo Fisher Scientific). Tandem mass spectra were searched against the amino-acid sequence of GST-α1^ins^-IL or Myc-α1^ins^. Trypsin was specified as the cleavage enzyme, allowing up to 2 missing cleavages. Mass error was set to 10 ppm for precursor ions and 0.02 Da for fragment ions. Serine/threonine phosphorylation and lysine ubiquitination were allowed as variable modifications.

### Immunohistochemistry

The mice were anesthetized with sodium pentobarbital at day 28 after viral injection and perfused through the ascending aorta with 4% paraformaldehyde in phosphate-buffered saline (PBS; 0.01 M). The lumbar enlargements of spinal cords were dissected out, fixed in the same fixative for 4 h, and cryoprotected in 30% sucrose overnight. The transverse or sagittal sections (16 μm) were cut on a cryostat, blocked with 10% normal goat serum (NGS) and 0.1% Triton X-100 in PBS for 12 h at 4 °C, and incubated with mouse anti-GFP or rabbit anti-c-fos antibody (Proteintech) at 4 °C for 72 h. To assay the synaptic distribution of α1^ins^, the transverse slices of 2-mm thickness were cut on a chopper at 4 °C and fixed with 4% paraformaldehyde for 30 min before cryoprotection [19,45]. The transverse slices (16 μm) were blocked and incubated with rabbit anti-α1^ins^ and mouse anti-gephyrin antibody (Synaptic System) at 4 °C for 12 h. After five washes with PBS, the slices were incubated with Alexa Fluor 488- and Cy3-conjugated secondary antibodies for 1 h before image capture with a confocal laser scanning microscope (FV1000; Olympus, Tokyo, Japan).

### Immunocytochemistry

HEK293T cells were transfected with Myc-α1^ins^, Myc-α1, or Myc-α3L. At 48 h after transfection, the cells were fixed with 4% paraformaldehyde and 4% sucrose in PBS for 15 min. After three washes with PBS, the cells were permeabilized in PBS containing 0.25% Triton X-100 for 15 min and blocked with 10% NGS in PBS overnight at 4 °C. GlyRs were labeled by anti-α1^ins^ antibody for 2 h at 4 °C. After five washes with PBS, the cells were stained for 1 h at room temperature with Cy3-conjugated secondary antibodies in 10% NGS-containing PBS.

The cultured spinal neurons at 10–12 days in vitro were co-transfected with shRNA-Eps15 or NC along with Myc-α1^ins^ and GlyR β subunit (β:α = 50:1) [46]. At 72 h after transfection, the neurons were treated with DHPG in culture media for 3 min at 37 °C. After washing DHPG out for three times with prewarmed culture media, the neurons were incubated in the media for 10 min at 37 °C, followed by washes with PBS containing 4% sucrose and fixation with 4% paraformaldehyde and 4% sucrose in PBS for 20 min. Surface receptors were labeled by mouse anti-Myc antibody for 2 h at room temperature. The neurons were washed with PBS containing 4% sucrose and blocked in PBS containing 0.25% Triton X-100 and 10% NGS for 30 min. Neurons were then stained with chicken anti-MAP2 antibody (Novus Biologicals, Littleton, CO, USA). After five washes with PBS, surface Myc proteins and MAP2 were visualized by incubation for 1 h at room temperature with Cy3-conjugated goat anti-mouse and Alexa Fluor 405-conjugated goat anti-chicken secondary antibody.

To assay the endocytosis, the cultured spinal neurons at 10–12 days in vitro were transfected with GlyR β subunit and Myc-α1^ins^ or Myc-α1^ins^(S380A). At 48 h after transfection, the surface-bound receptors were labeled with mouse anti-Myc antibody for 2 h at 4 °C. The neurons were washed 3 times with prewarmed culture media and treated with DHPG for 3 min at 37 °C. After washing DHPG out with prewarmed culture media, the neurons were maintained in the incubator for 10 min. Thereafter, the neurons were washed twice with ice-cold PBS and fixed with PBS containing 4% paraformaldehyde and 4% sucrose for 20 min at room temperature. The remaining surface anti-Myc antibody was visualized by staining with Alexa Fluor 488-conjugated anti-mouse IgG at room temperature for 1 h. The cells were then permeabilized and blocked in PBS containing 0.25% Triton X-100 and 10% NGS for 30 min. The cells were stained with chicken anti-MAP2 antibody. After five washes with PBS, the internalized anti-Myc antibody and MAP2 were visualized by staining with Cy3-conjugated anti-mouse and Alexa Fluor 405-conjugated anti-chicken IgG before image capture. The internalization signals were divided by the signals of surface plus internalized receptors.

### Behavioral tests

The animals were acclimatized to the testing environment for at least 1 h before intraplantar formalin or intrathecal DHPG injection. Immediately after the injection, we returned the animals to the chamber and observed the spontaneous pain behaviors for 0.5–1 h [47,48]. For the Von Frey test, a set of calibrated Von Frey monofilaments (Stoelting, Wood Dale, IL, USA) were applied perpendicularly to the plantar surfaces of hindpaws. The pattern of positive and negative withdrawal responses was converted to 50% paw withdrawal thresholds by using the up–down method [20]. The paw withdrawal latencies (PWLs) were measured by delivering a beam of light onto the plantar surfaces of hindpaws (with the cutoff of 10 s). The time between the onset of heat application and paw withdrawal was recorded automatically as PWL values [20]. Cold stimulation was delivered by dabbing acetone onto the plantar surfaces of hindpaws. The first 10-second activities were excluded, and the time spent on flicking and licking the paws for 60 s afterwards was recorded [49]. Motor function was tested on a rotarod that was accelerated from 0 to 40 RPM within 60 s. After training for two days, the mice were tested on the rod for three times, and the maximum RPM that caused the mice to fall was averaged [3].

### Electrophysiological recordings

The mice (6–8 weeks old) were anesthetized with sodium pentobarbital, and the lumbar segment of spinal cord was isolated into ice-cold sucrose solution (in mM: 50.0 sucrose, 95.0 NaCl, 1.8 KCl, 0.5 CaCl_2_, 7.0 MgSO_4_, 1.2 NaH_2_PO_4_, 26.0 NaHCO_3_, 15.0 D-glucose [pH 7.4], bubbled with 95% O_2_ + 5% CO_2_). A transverse slice (350-μm thickness) with an intact L4 or L5 dorsal root was cut on a vibratome stage and perfused (5 ml/min) with oxygenated ACSF (32 °C–33 °C) in the recording chamber for at least 1 h before recordings. The lamina II outer neurons were visually identified under an Olympus BX51WIF microscope fitted with a 40× water immersion objective under fluorescence and transmitted light illumination. We performed the recordings on lamina II outer neurons because these neurons receive the inputs from unmyelinated peptidergic C nociceptors and myelinated Aδ nociceptors as well as from spinal glycinergic inhibitory interneurons [3,50–52]. The reduced glycinergic inhibition contributes to the development of pathological pain [3,50]. The glass electrodes had the resistance of 3–5 MΩ when filled with the internal solution (in mM: 110.0 Cs_2_SO_4_, 5.0 KCl, 2.0 MgCl_2_, 0.5 CaCl_2_, 5.0 HEPES, 5.0 EGTA, 5.0 Mg-ATP, and 0.5 Na-GTP [pH 7.25]; 295–300 mOsm). The neurons were voltage-clamped at 0 mV with an Axon 700B amplifier (Molecular Devices, San Jose, CA, USA). To evoke GlyR-IPSCs, focal stimulation (0.1 Hz) was delivered through a glass pipette that was positioned adjacent to the recorded neurons [15]. The glycinergic component was pharmacologically isolated by adding bicuculline (10 μM), D-APV (50 μM), and CNQX (20 μM) in the external solution. The paired-pulse ratios were recorded and measured by delivering two successive electric stimuli at a 30-ms interval. For mIPSCs, tetrodotoxin (1 μM) was also included in the bath solution. The GABAergic IPSCs were isolated by strychnine (2 μM), D-APV, and CNQX. To record the dorsal-root–evoked EPSCs, the glass pipettes were filled with (in mM) 115 cesium methanesulfonate, 20 CsCl, 10 HEPES, 2.5 MgCl_2_, 4.0 Na_2_ATP, 0.4 Na-GTP, 0.6 EGTA, and 10 sodium phosphocreatine (pH 7.25; 295–300 mOsm). NMDAR-EPSCs were recorded at +40 mV in the presence of bicuculline, strychnine, and CNQX. The AMPAR-EPSCs were recorded at −70 mV in the presence of bicuculline and strychnine. The monosynaptic EPSCs were identified on the basis of the constant latency and absence of conduction failure in response to high-frequency electrical stimulation (20 Hz). To evoke the whole-cell glycinergic currents in spinal slices [15], glycine (1 mM, 5 s) was perfused onto the recorded neurons through an electrically controlled microperfusion system. HEK293T cells co-transfected with pcDNA3.1 and pEGFP-N1 vector (reporter plasmid) were perfused at room temperature with the external solution containing (mM) 145.0 NaCl, 5.0 KCl, 2.0 CaCl_2_, 1.0 MgCl_2_, 10.0 HEPES, and 11.0 D-glucose (pH 7.3) [19]. When filled with the internal solution (in mM: 140.0 CsCl, 1.0 CaCl_2_, 2.0 MgCl_2_, 10.0 HEPES, 8.0 EGTA, 3.0 Na-ATP, and 0.1 Na-GTP [pH 7.2]; 295–300 mOsm), the recording pipettes had the resistance of 3–5 MΩ. The cells were voltage-clamped at −80 mV. To elicit the whole-cell glycinergic currents, glycine (1 mM, 10 ms) was dissolved in the external solution and rapidly applied onto the cells at an interval of 30 s. The series and input resistances were monitored online throughout each experiment. The recordings were collected for analysis unless the resistances changed by more than 15%. The current signals were filtered at 2 kHz and sampled at 5 kHz.

### Statistics

We presented the data as mean ± SEM. The behavioral tests, GST pull-down, electrophysiological recordings, immunocytochemistry, and phosphorylation assay were conducted by the investigators unaware of the group allocation. The data were randomly collected and processed. The synaptic responses were electrically evoked at 0.1 Hz, the consecutive 6 responses every minute were averaged, and the peak amplitudes were analyzed by Clampfit 8.0 software. The mIPSC signals were analyzed by Mini-analysis software. Western blot data were quantified by NIH ImageJ software. For immunocytochemistry, one 30- to 50-μm dendritic segment was randomly selected from each neuron, and the Image-Pro Plus 6.0 software was used to analyze the immunofluorescence intensities. Two group comparisons were conducted by using paired Student *t* test or Mann–Whitney U test. One-way Analysis of Variance (ANOVA) followed by post hoc Bonferroni test was used for the data across multiple groups. The repeated measurement ANOVA and Bonferroni post hoc tests were used to compare the data between multiple groups occurring over time. The criterion for statistical significance was *p* < 0.05.

## Supporting information

S1 Data Excel spreadsheet containing the numerical data for Figure panels 1A, 1B, 1C, 1D, 1E, 1F, 1G, 1H, 2A, 2B, 3B, 3C, 3D, 3E, 3F, 3G, 3H, 3I, 4D, 4G, 4H, 4I, 4J, 4K, 5A, 5C, 5E, 5F, 5G, 5H, 6A, 6B, 6D, 6E, 6H, 7A, 7B, 7C, 7D, 7E, 7F, S1, S2, S4, S5, S6, S7, and S8. (XLSX)

S1 Fig Bath application of DHPG for 3 min reduced GlyR-IPSCs when the spinal slices of mice were pretreated with CB1 receptor blocker AM251 for 30 min (74.9 ± 7.2% of baseline at 15–20 min post-DHPG, *t*[5] = 3.841, *p* = 0.012, paired Student *t* test). The horizontal bar indicated the period of DHPG or AM251 perfusion. The original traces were taken at the time points indicated by the numbers 1–3. The underlying data for this figure can be found in S1 Data. Error bars indicated SEM. AM251, 1-(2,4-Dichlorophenyl)-5-(4-iodophenyl)-4-methyl-N-1-piperidinyl-1H-pyrazole-3-carboxamide; CB1, Type-1 cannabinoid; DHPG, (S)-3,5-Dihydroxyphenylglycine; GlyR, glycine receptor; IPSC, inhibitory postsynaptic current.
(TIF)

S2 Fig The inhibitory effect of DHPG on α1^ins^ currents in transfected HEK293T cells was blocked by intracellular loading of PD98059 (87.6 ± 6.3% of baseline at 10–15 min post-DHPG, *t*[5] = 1.48, *p* = 0.199) but not by Ro-32-0432 (66.5 ± 9.1% of baseline at 10–15 min post-DHPG, *t*[5] = 2.743, *p* = 0.041). The horizontal bar indicated the period of DHPG perfusion. The underlying data for this figure can be found in S1 Data. Error bars indicated SEM. DHPG, (S)-3,5-Dihydroxyphenylglycine; HEK, human embryonic kidney; PD98059, 2′-Amino-3′-methoxyflavone; Ro-32-0432, 2-{8-[(Dimethylamino)methyl]-6,7,8,9-tetrahydropyrido[1,2-a]indol-3-yl}-3-(1-methyl-1H-indol-3-yl)maleimide.
(TIF)

S3 Fig Specificity of anti-α1^ins^ antibody. (**A–B**) Immunofluorescent (**A**) and western blot analysis (**B**) of transfected HEK293T cells using anti-α1^ins^ antibody. The cells were transfected with α1^ins^, α1, or α3L. Preincubation with excess antigen abolished anti-α1^ins^ signals (**B**). Scale bar: 10 μm. (**C**) Double immunofluorescence for α1^ins^ (red) and gephyrin (green) in the dorsal horn of spinal cord. Preincubation with excess antigen attenuated anti-α1^ins^ signals. *n* = 6 slices from 2 mice/group. Scale bar: 5 μm. HEK, human embryonic kidney.
(TIF)

S4 Fig Specificity of shRNA against mouse α1^ins^ (shRNA-α1^ins^). (**A**) α1^ins^ or α1 was co-transfected with shRNA-α1^ins^ in HEK293T cells and probed with anti-α1 antibody at day 3 post-transfection. A negative shRNA was used as control (NC). *p < 0.001 versus NC (Mann–Whitney U test), *n* = 6. (**B**) GFP fluorescence spread rostrocaudally for about 0.5 mm from the site where AAV encoding GFP and shRNA-α1^ins^ was injected. Scale bar: 0.5 mm. (**C**) Intraspinal injection of AAV encoding shRNA-α1^ins^ decreased the protein level of α1^ins^ but not of α3. *p = 0.017 versus NC (one-way ANOVA with post hoc Bonferroni test), *n* = 6. The underlying data for this figure can be found in S1 Data. Error bars indicated SEM. AAV, adeno-associated virus; ANOVA, Analysis of Variance; GFP, green fluorescent protein; HEK, human embryonic kidney; NC, negative control shRNA; shRNA, short hairpin RNA.
(TIF)

S5 Fig Intraspinal injection of AAV encoding shRNA against mouse α3 (shRNA-α3) elicited painful behaviors but did not block the inhibitory effect of DHPG on GlyR-IPSCs in mice. (**A**) shRNA-α3 specifically decreased the protein level of α3. *p < 0.001 versus NC (one-way ANOVA with post hoc Bonferroni test), *n* = 6. (**B–D**) shRNA-α3 evoked mechanical allodynia (**B**, F[4, 72] = 7.905, *p* < 0.001, repeated measures ANOVA, *n* = 10 mice/group), heat hyperalgesia (**C**, F[4, 72] = 3.331, *p* = 0.015, *n* = 10 mice/group), and cold hyperalgesia (**D**, F[4, 56] = 4.164, *p* = 0.005, *n* = 8 mice/group). (**E**) shRNA-α3 did not block DHPG from inhibiting GlyR-IPSCs (71.5 ± 4.2% of baseline at 15–20 min post-DHPG, *t*[5] = 5.231, *p* = 0.003, paired Student *t* test). The underlying data for this figure can be found in S1 Data. Error bars indicated SEM. AAV, adeno-associated virus; ANOVA, Analysis of Variance; DHPG, (S)-3,5-Dihydroxyphenylglycine; GlyR, glycine receptor; IPSC, inhibitory postsynaptic current; NC, negative control shRNA; shRNA, short hairpin RNA.
(TIF)

S6 Fig shRNA knockdown of Eps15 blocked DHPG from decreasing the surface expression of Myc-α1^ins^ in cultured neurons. (**A**) Eps15 protein level at day 3 after transfection of shRNA-Eps15 or NC. *p = 0.014 versus NC (one-way ANOVA with post hoc Bonferroni test), *n* = 4. (**B**) Immunostaining of surface Myc-α1^ins^ (red) and MAP2 (blue) in NC- or shRNA-Eps15–transfected neurons with or without DHPG (10 μM) treatment. *p < 0.001 versus control (one-way ANOVA with post hoc Bonferroni test), *n* = 30 neurons/group. Scale bar, 5 μm. The underlying data for this figure can be found in S1 Data. Error bars indicated SEM. ANOVA, Analysis of Variance; DHPG, (S)-3,5-Dihydroxyphenylglycine; Eps15, epidermal growth factor receptor substrate 15; MAP2, Microtubule-Associated Protein 2; NC, negative control shRNA; shRNA, short hairpin RNA.
(TIF)

S7 Fig Effects of TAT-pep-α1^ins^ and TAT-Scram (200 pmol) on the mechanical allodynia induced by SNI. The arrow indicated the time point when peptides were intrathecally injected at day 14 post-SNI. F(6, 108) = 7.862, *p* < 0.001 (repeated measures ANOVA). *n* = 10 mice/group. The underlying data for this figure can be found in S1 Data. Error bars indicated SEM. ANOVA, Analysis of Variance; SNI, spared nerve injury; TAT-pep-α1^ins^, TAT-fused α1^ins^-derived peptide; TAT-Scram, TAT-fused scrambled peptide.
(TIF)

S8 Fig TAT-pep-α1^ins^ specifically potentiated GlyR-IPSCs in spinal dorsal horn of formalin-injected mice. (**A–B**) Viral expression of shRNA-α1^ins^ blocked TAT-pep-α1^ins^ from potentiating GlyR-IPSCs (**A**, 109.5 ± 14.6% of baseline at 15–20 min postpeptide, *t*[6] = 0.873, *p* = 0.416, paired Student *t* test), whereas shRNA-α3 had no effect (**B**, 146.9 ± 13.0% of baseline at 15–20 min postpeptide, *t*[5] = 3.833, *p* = 0.012). (**C–E**) TAT-pep-α1^ins^ did not affect the synaptic transmission mediated by AMPAR (**C**, 95.7 ± 5.2% of baseline at 15–20 min postpeptide, *t*[5] = 0.685, *p* = 0.524), NMDAR (**D**, 101.9 ± 7.1% of baseline at 15–20 min postpeptide, *t*[6] = 0.743, *p* = 0.485), or GABA_A_R (**E**, 100.8 ± 6.9% of baseline at 15–20 min postpeptide, *t*[5] = 0.388, *p* = 0.714) in formalin-injected mice. (**F**) GlyR-IPSCs in intact mice were insensitive to TAT-pep-α1^ins^ (111.1 ± 10.2% of baseline at 15–20 min postpeptide, *t*[5] = 0.656, *p* = 0.541). The underlying data for this figure can be found in S1 Data. Error bars indicated SEM. AMPAR, α-Amino-3-hydroxy-5-methylisoxazole-4-propionic Acid receptor; GABA_A_R, γ-Aminobutyric acid type A receptor; GlyR, glycine receptor; IPSC, inhibitory postsynaptic current; NMDAR, N-methyl-D-aspartate receptor; shRNA, short hairpin RNA; TAT-pep-α1^ins^, TAT-fused α1^ins^-derived peptide.
(TIF)

## Abbreviations

AAV: adeno-associated virus
ACSF: artificial cerebrospinal fluid
AMPA: α-Amino-3-hydroxy-5-methylisoxazole-4-propionic Acid
ANOVA: Analysis of Variance
cAMP: cyclic adenosine monophosphate
CB1: Type-1 cannabinoid
CHPG: α-amino-2-chloro-5-hydroxybenzeneacetic acid
DHPG: (S)-3,5-Dihydroxyphenylglycine
DMEM: Dulbecco’s modified Eagle’s medium
Eps15: epidermal growth factor receptor substrate 15
ERK: extracellular signal-regulated kinase
GABA_A_ receptor: γ-Aminobutyric acid type A receptor
GDP-β-S: Guanosine 5′-O-(2-Thiodiphosphate)
GFP: green fluorescent protein
GlyR: glycine receptor
GST: Glutathione S-Transferase
HEK: human embryonic kidney
HUWE1: HECT, UBA, WWE domain contain 1
IgG: immunoglobulin G
IL: intracellular large loop
IPSC: inhibitory postsynaptic current
LC MS/MS: liquid chromatograph/mass spectrometer
MAP2: Microtubule-Associated Protein 2
MEK: mitogen-activated protein kinase kinase
mGluR5: metabotropic glutamate receptor 5
mIPSC: miniature IPSC
MOPS: morpholinepropanesulfonic acid
MPEP: 6-Methyl-2-(phenylethynyl) pyridine
NC: negative control shRNA
NGS: normal goat serum
NMDA: N-methyl-D-aspartate
PBS: phosphate-buffered saline
PD98059: 2ʹ-Amino-3ʹ-methoxyflavone
*p*ERK: phosphorylated ERK
PKA: cAMP-dependent protein kinase
PKC: Protein Kinase C
*p*S380: phosphorylated α1^ins^ at Ser380
pS380-Ab: phosphorylation-state–specific antibody against Ser380 on α1^ins^
PWL: paw withdrawal latency
RIPA: radio-immunoprecipitation assay
Ro-32-0432: 2-{8-[(Dimethylamino)methyl]-6,7,8,9-tetrahydropyrido[1,2-a]indol-3-yl}-3-(1-methyl-1H-indol-3-yl)maleimide
RPM: rounds per minute
SDS-PAGE: SDS-Polyacrylamide Gel Electrophoresis
shRNA: short hairpin RNA
SP: serine–proline motif
TAT: human immunodeficiency virus-type 1 TAT sequence
TAT-pep-α1^ins^: TAT-fused α1^ins^-derived peptide
TAT-Scram: TAT-fused scrambled peptide
TCL: total cell lysate
TM: transmembrane
Ubi: ubiquitin
