# Supplementary figures and images for: mGluR5/ERK signaling regulated the phosphorylation and function of glycine receptor α1ins subunit in spinal dorsal horn of mice
Source: PLoS Biol. 2019 Aug 21;17(8):e3000371. doi: 10.1371/journal.pbio.3000371 (PMC6703679; doi:10.1371/journal.pbio.3000371)

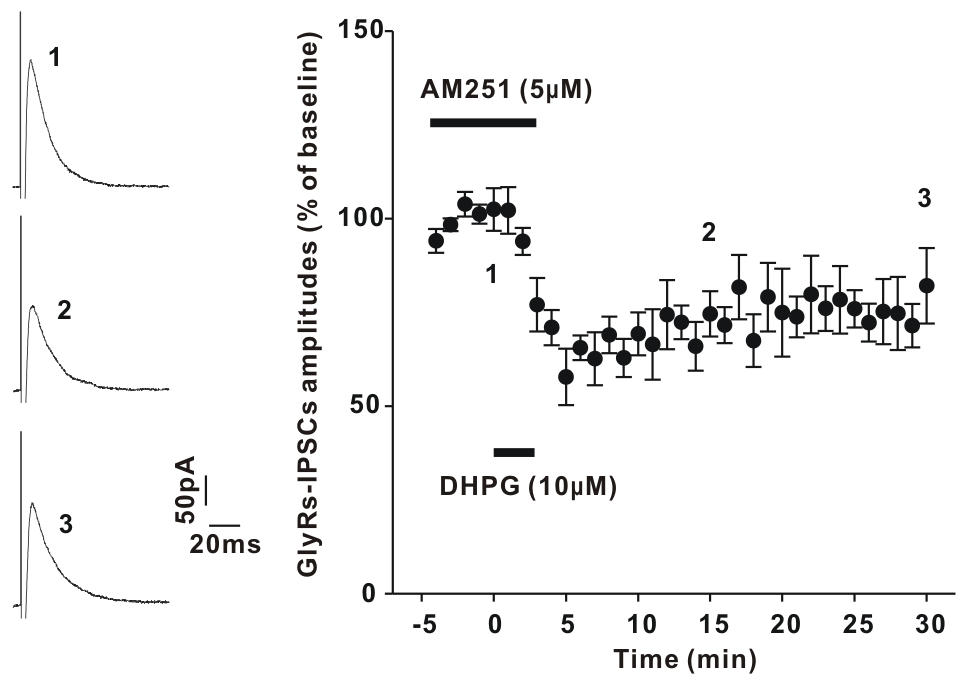

Supplement: S1 Fig — The horizontal bar indicated the period of DHPG or AM251 perfusion. The original traces were taken at the time points indicated by the numbers 1–3. The underlying data for this figure can be found in S1 Data. Error bars indicated SEM. AM251, 1-(2,4-Dichlorophenyl)-5-(4-iodophenyl)-4-methyl-N-1-piperidinyl-1H-pyrazole-3-carboxamide; CB1, Type-1 cannabinoid; DHPG, (S)-3,5-Dihydroxyphenylglycine; GlyR, glycine receptor; IPSC, inhibitory postsynaptic current. (TIF) [file pbio.3000371.s002.tif]

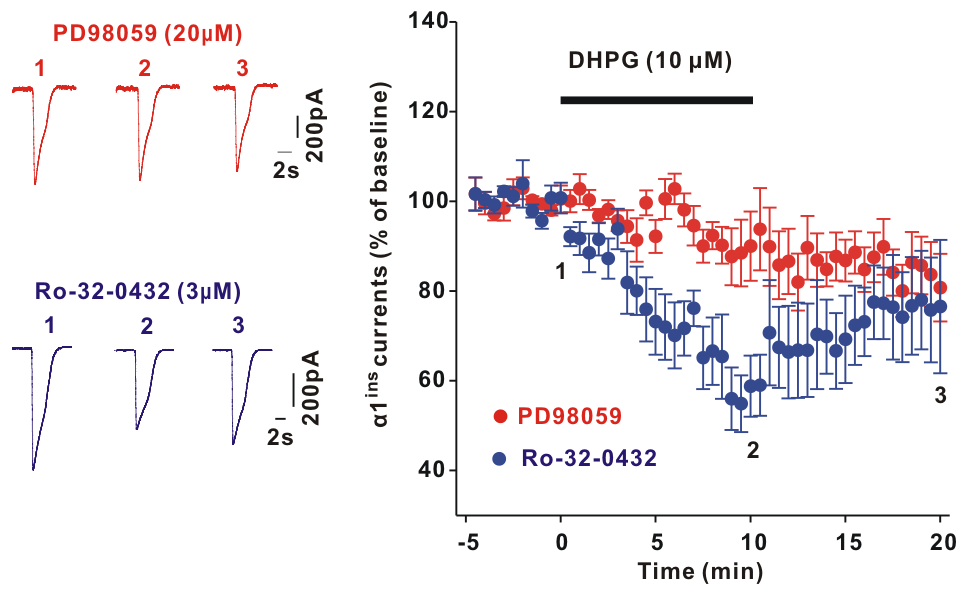

Supplement: S2 Fig — The horizontal bar indicated the period of DHPG perfusion. The underlying data for this figure can be found in S1 Data. Error bars indicated SEM. DHPG, (S)-3,5-Dihydroxyphenylglycine; HEK, human embryonic kidney; PD98059, 2′-Amino-3′-methoxyflavone; Ro-32-0432, 2-{8-[(Dimethylamino)methyl]-6,7,8,9-tetrahydropyrido[1,2-a]indol-3-yl}-3-(1-methyl-1H-indol-3-yl)maleimide. (TIF) [file pbio.3000371.s003.tif]

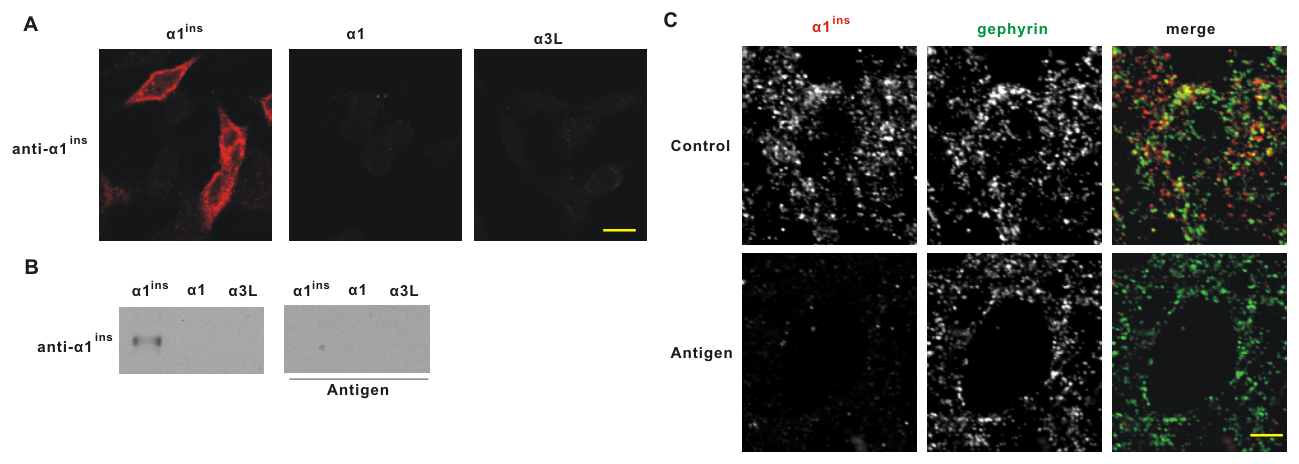

Supplement: S3 Fig — (A–B) Immunofluorescent (A) and western blot analysis (B) of transfected HEK293T cells using anti-α1ins antibody. The cells were transfected with α1ins, α1, or α3L. Preincubation with excess antigen abolished anti-α1ins signals (B). Scale bar: 10 μm. (C) Double immunofluorescence for α1ins (red) and gephyrin (green) in the dorsal horn of spinal cord. Preincubation with excess antigen attenuated anti-α1ins signals. n = 6 slices from 2 mice/group. Scale bar: 5 μm. HEK, human embryonic kidney. (TIF) [file pbio.3000371.s004.tif]

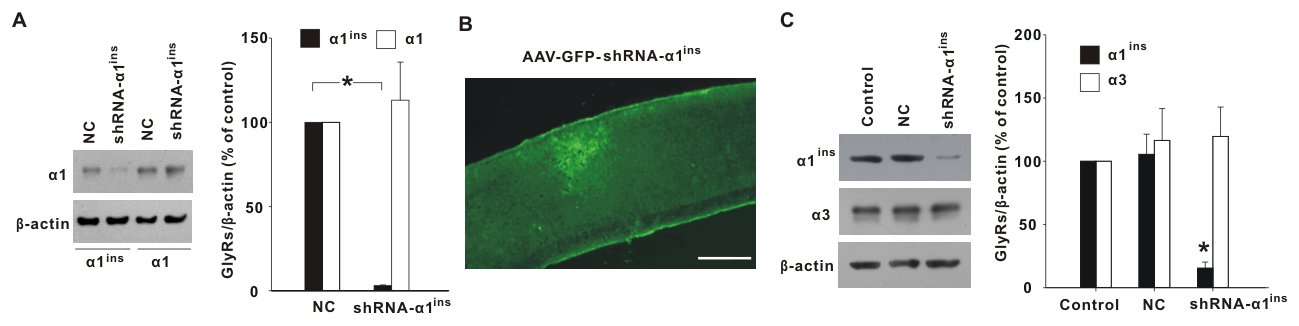

Supplement: S4 Fig — (A) α1ins or α1 was co-transfected with shRNA-α1ins in HEK293T cells and probed with anti-α1 antibody at day 3 post-transfection. A negative shRNA was used as control (NC). *p < 0.001 versus NC (Mann–Whitney U test), n = 6. (B) GFP fluorescence spread rostrocaudally for about 0.5 mm from the site where AAV encoding GFP and shRNA-α1ins was injected. Scale bar: 0.5 mm. (C) Intraspinal injection of AAV encoding shRNA-α1ins decreased the protein level of α1ins but not of α3. *p = 0.017 versus NC (one-way ANOVA with post hoc Bonferroni test), n = 6. The underlying data for this figure can be found in S1 Data. Error bars indicated SEM. AAV, adeno-associated virus; ANOVA, Analysis of Variance; GFP, green fluorescent protein; HEK, human embryonic kidney; NC, negative control shRNA; shRNA, short hairpin RNA. (TIF) [file pbio.3000371.s005.tif]

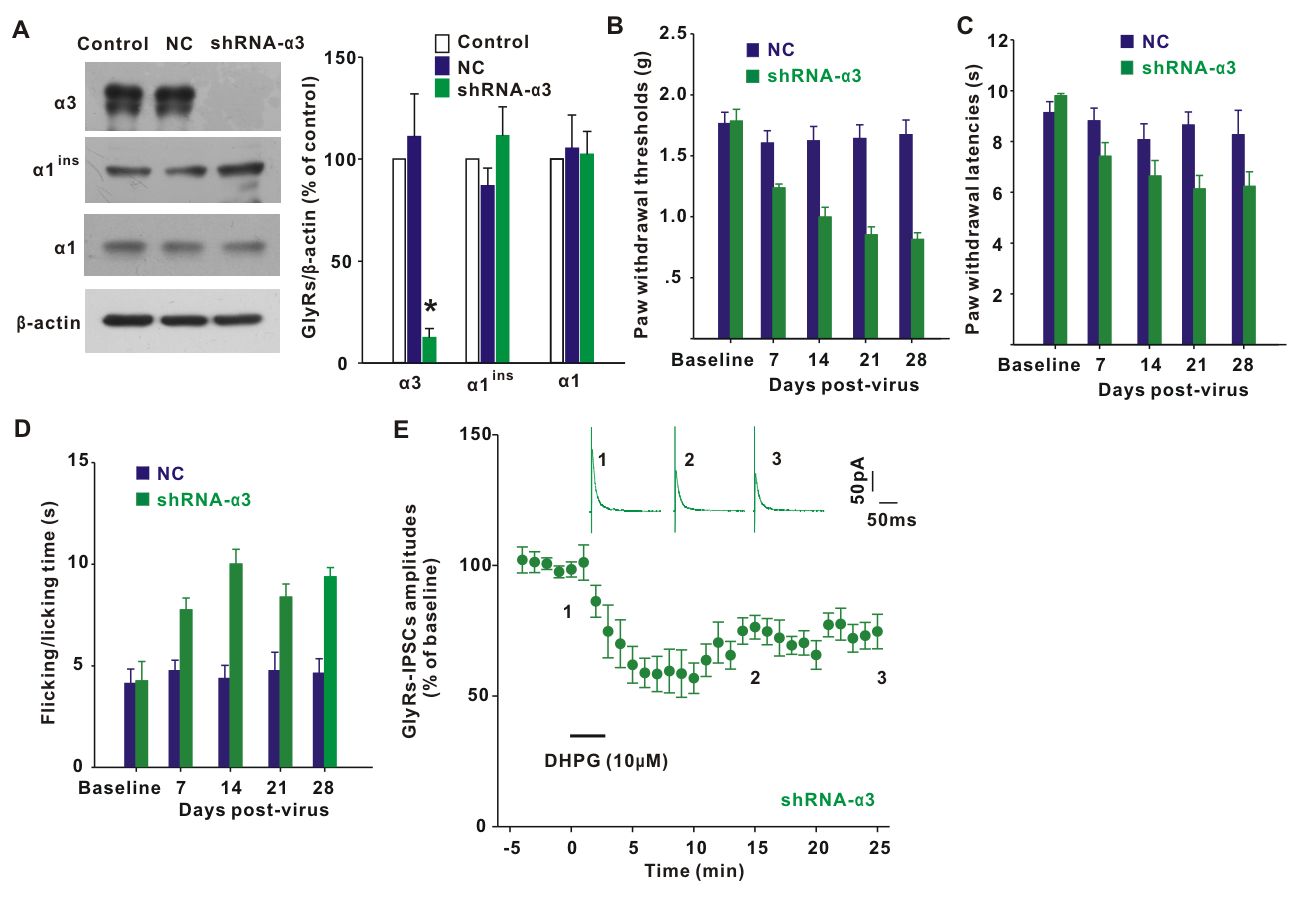

Supplement: S5 Fig — (A) shRNA-α3 specifically decreased the protein level of α3. *p < 0.001 versus NC (one-way ANOVA with post hoc Bonferroni test), n = 6. (B–D) shRNA-α3 evoked mechanical allodynia (B, F[4, 72] = 7.905, p < 0.001, repeated measures ANOVA, n = 10 mice/group), heat hyperalgesia (C, F[4, 72] = 3.331, p = 0.015, n = 10 mice/group), and cold hyperalgesia (D, F[4, 56] = 4.164, p = 0.005, n = 8 mice/group). (E) shRNA-α3 did not block DHPG from inhibiting GlyR-IPSCs (71.5 ± 4.2% of baseline at 15–20 min post-DHPG, t[5] = 5.231, p = 0.003, paired Student t test). The underlying data for this figure can be found in S1 Data. Error bars indicated SEM. AAV, adeno-associated virus; ANOVA, Analysis of Variance; DHPG, (S)-3,5-Dihydroxyphenylglycine; GlyR, glycine receptor; IPSC, inhibitory postsynaptic current; NC, negative control shRNA; shRNA, short hairpin RNA. (TIF) [file pbio.3000371.s006.tif]

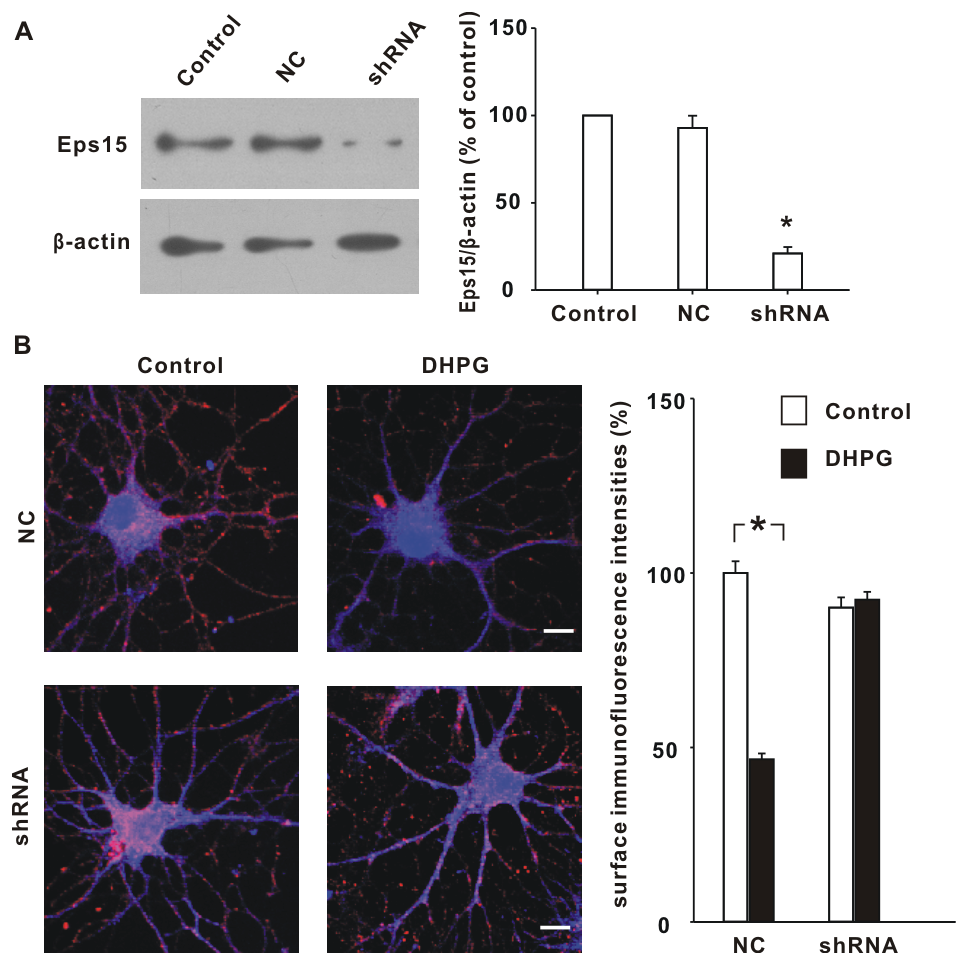

Supplement: S6 Fig — (A) Eps15 protein level at day 3 after transfection of shRNA-Eps15 or NC. *p = 0.014 versus NC (one-way ANOVA with post hoc Bonferroni test), n = 4. (B) Immunostaining of surface Myc-α1ins (red) and MAP2 (blue) in NC- or shRNA-Eps15–transfected neurons with or without DHPG (10 μM) treatment. *p < 0.001 versus control (one-way ANOVA with post hoc Bonferroni test), n = 30 neurons/group. Scale bar, 5 μm. The underlying data for this figure can be found in S1 Data. Error bars indicated SEM. ANOVA, Analysis of Variance; DHPG, (S)-3,5-Dihydroxyphenylglycine; Eps15, epidermal growth factor receptor substrate 15; MAP2, Microtubule-Associated Protein 2; NC, negative control shRNA; shRNA, short hairpin RNA. (TIF) [file pbio.3000371.s007.tif]

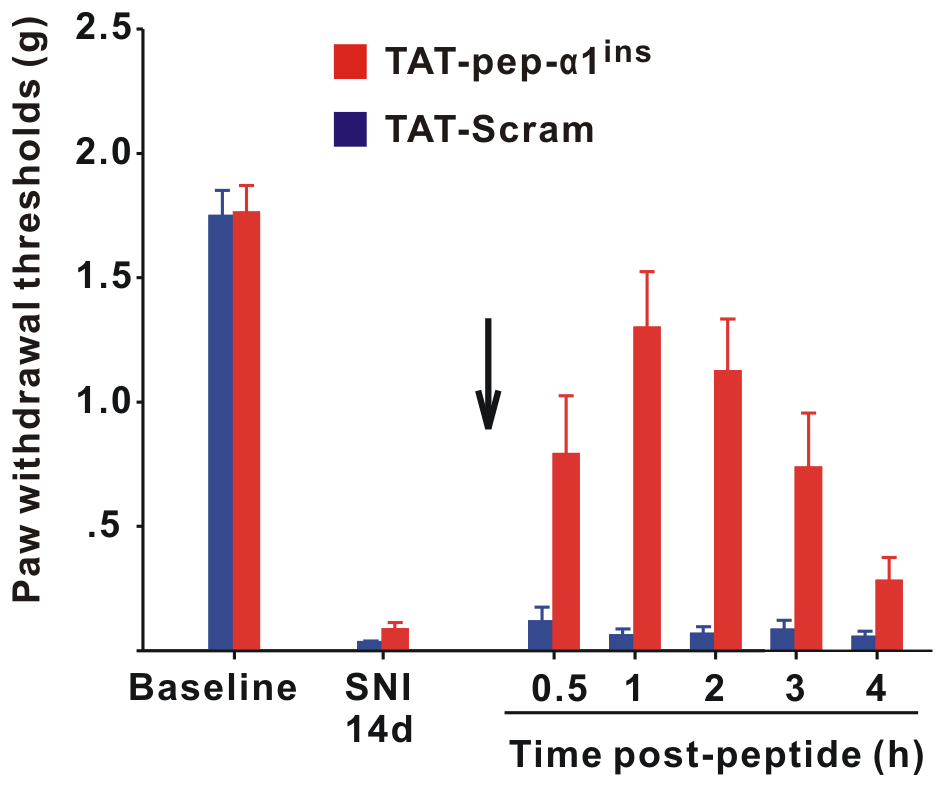

Supplement: S7 Fig — The arrow indicated the time point when peptides were intrathecally injected at day 14 post-SNI. F(6, 108) = 7.862, p < 0.001 (repeated measures ANOVA). n = 10 mice/group. The underlying data for this figure can be found in S1 Data. Error bars indicated SEM. ANOVA, Analysis of Variance; SNI, spared nerve injury; TAT-pep-α1ins, TAT-fused α1ins-derived peptide; TAT-Scram, TAT-fused scrambled peptide. (TIF) [file pbio.3000371.s008.tif]

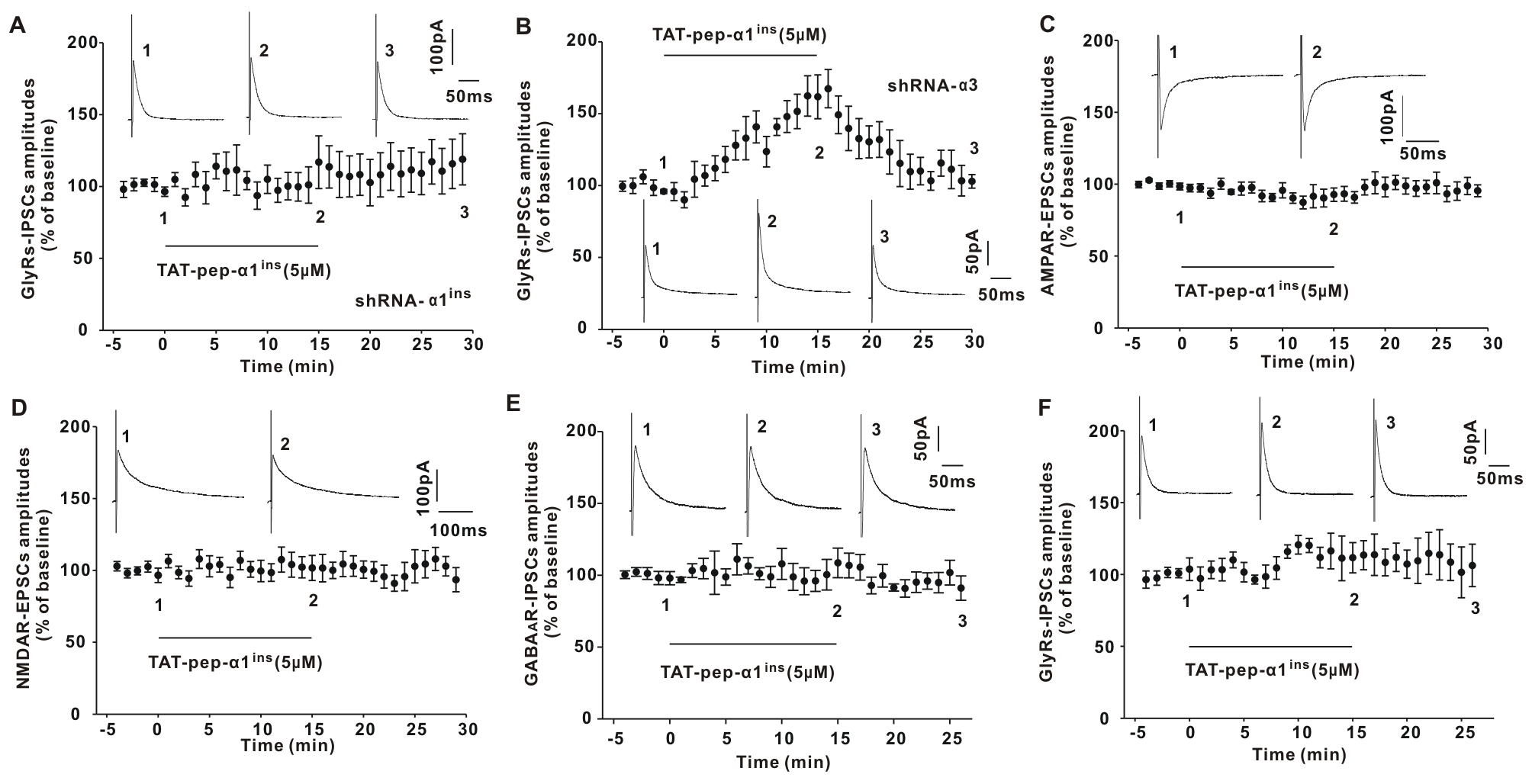

Supplement: S8 Fig — (A–B) Viral expression of shRNA-α1ins blocked TAT-pep-α1ins from potentiating GlyR-IPSCs (A, 109.5 ± 14.6% of baseline at 15–20 min postpeptide, t[6] = 0.873, p = 0.416, paired Student t test), whereas shRNA-α3 had no effect (B, 146.9 ± 13.0% of baseline at 15–20 min postpeptide, t[5] = 3.833, p = 0.012). (C–E) TAT-pep-α1ins did not affect the synaptic transmission mediated by AMPAR (C, 95.7 ± 5.2% of baseline at 15–20 min postpeptide, t[5] = 0.685, p = 0.524), NMDAR (D, 101.9 ± 7.1% of baseline at 15–20 min postpeptide, t[6] = 0.743, p = 0.485), or GABAAR (E, 100.8 ± 6.9% of baseline at 15–20 min postpeptide, t[5] = 0.388, p = 0.714) in formalin-injected mice. (F) GlyR-IPSCs in intact mice were insensitive to TAT-pep-α1ins (111.1 ± 10.2% of baseline at 15–20 min postpeptide, t[5] = 0.656, p = 0.541). The underlying data for this figure can be found in S1 Data. Error bars indicated SEM. AMPAR, α-Amino-3-hydroxy-5-methylisoxazole-4-propionic Acid receptor; GABAAR, γ-Aminobutyric acid type A receptor; GlyR, glycine receptor; IPSC, inhibitory postsynaptic current; NMDAR, N-methyl-D-aspartate receptor; shRNA, short hairpin RNA; TAT-pep-α1ins, TAT-fused α1ins-derived peptide. (TIF) [file pbio.3000371.s009.tif]
